# Supplementary figures and images for: STUPPIT is a proximity labeling tool for labeling intermediary proteins that bridge two non-interacting proteins
Source: PLoS Biol. 2025 Nov 24;23(11):e3003227. doi: 10.1371/journal.pbio.3003227 (PMC12668627; doi:10.1371/journal.pbio.3003227)

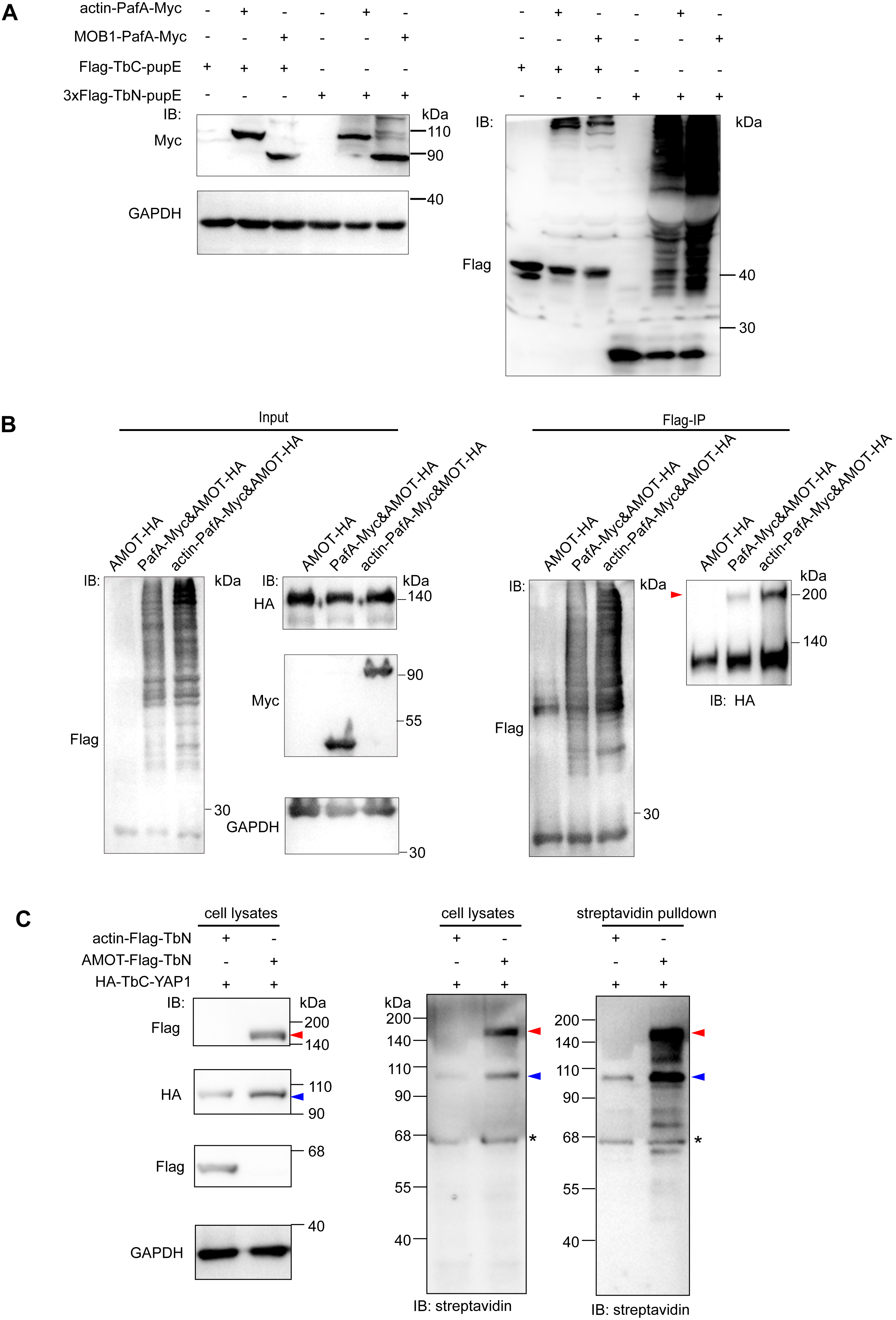

Supplement: S1 Fig — (A) Validation of the labeling efficiency of TbC-PupE and TbN-PupE on interacting proteins by PafA. (B) Immunoblotting confirming that actin-PafA-Myc ligates the 3 × Flag-TbN-PupE substrate onto exogenous AMOT-HA. 3 × Flag-TbN-PupE stably expressed cells were transfected with AMOT-HA alone, PafA-Myc&AMOT-HA, or actin-PafA-Myc&AMOT-HA. The red arrowhead indicates the molecular weight laddering of AMOT-HA after Flag-IP. (C) Split-TurboID between actin-Flag-TbN and HA-TbC-YAP1, AMOT-Flag-TbN and HA-TbC-YAP1. The red arrowheads indicate size of AMOT-Flag-TbN, and blue arrowheads indicate size of HA-TbC-YAP1, while asterisks represent non-specific bands. (TIF) [file pbio.3003227.s001.tif]

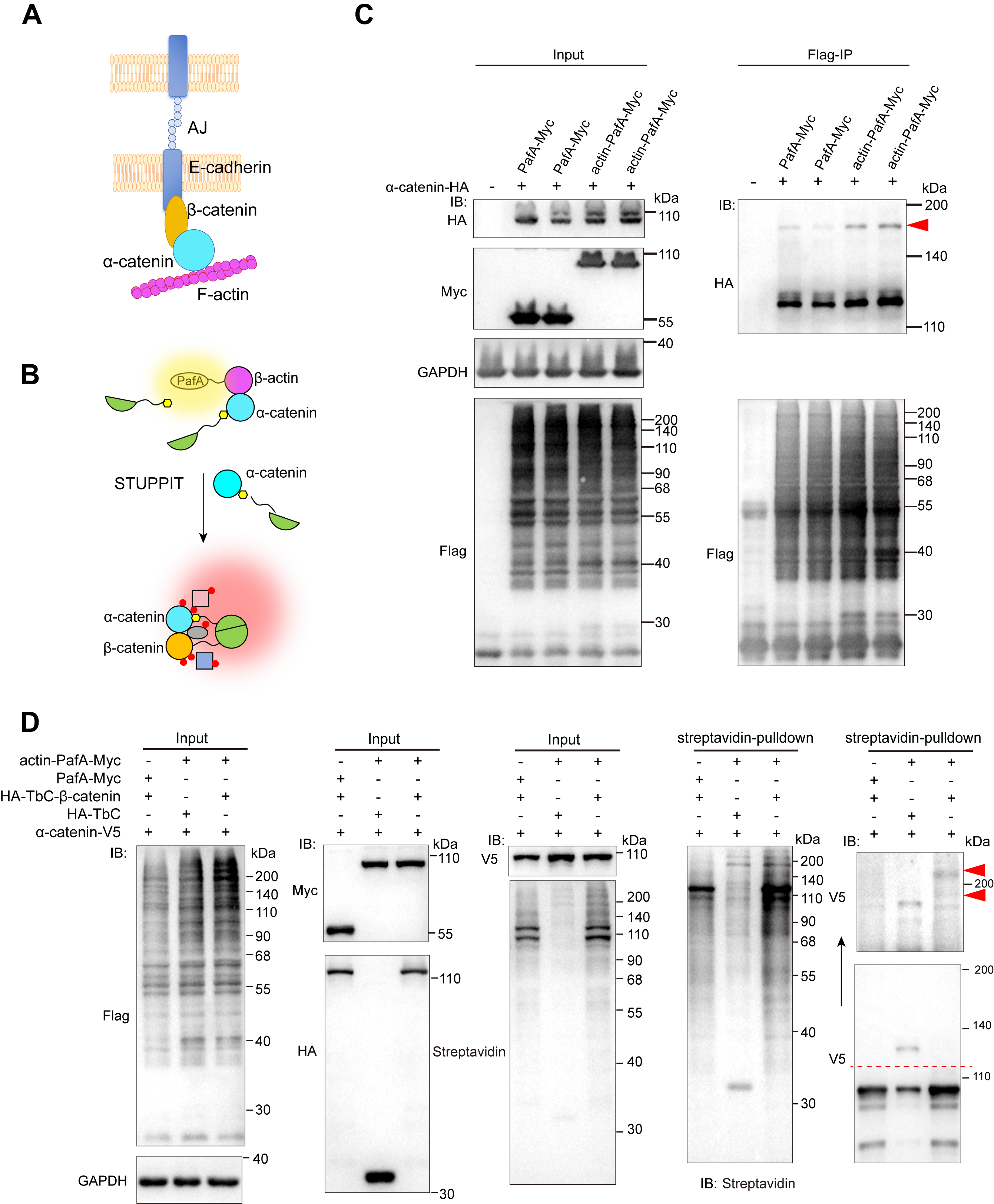

Supplement: S2 Fig — (A) α-catenin serves as the intermediary protein linking β-catenin and actin. (B) Schematic representation of the labeling of the intermediary protein α-catenin between actin and β-catenin using STUPPIT. (C) Validation of the conjugation of substrate 3 × Flag-TbN-PupE to α-catenin-HA by actin-PafA-Myc through immunoblotting. 3 × Flag-TbN-PupE stably expressed cells were transfected with α-catenin-HA&PafA-Myc, or α-catenin-HA&actin-PafA-Myc. The red solid arrows indicate the molecular weight laddering of α-catenin-HA in actin-PafA-Myc transfected cells after Flag-IP. (D) Validation of the intermediary protein α-catenin between β-catenin and actin captured by STUPPIT through immunoblotting. 3 × Flag-TbN-PupE stably expressed cells were transfected with α-catenin-V5&PafA-Myc&TbC-β-catenin, α-catenin-V5&actin-PafA-Myc&HA-TbC, or α-catenin-V5&actin-PafA-Myc&TbC-β-catenin. The red solid arrow indicates the molecular weight laddering of α-catenin-V5 after the streptavidin-pulldown. (TIF) [file pbio.3003227.s002.tif]

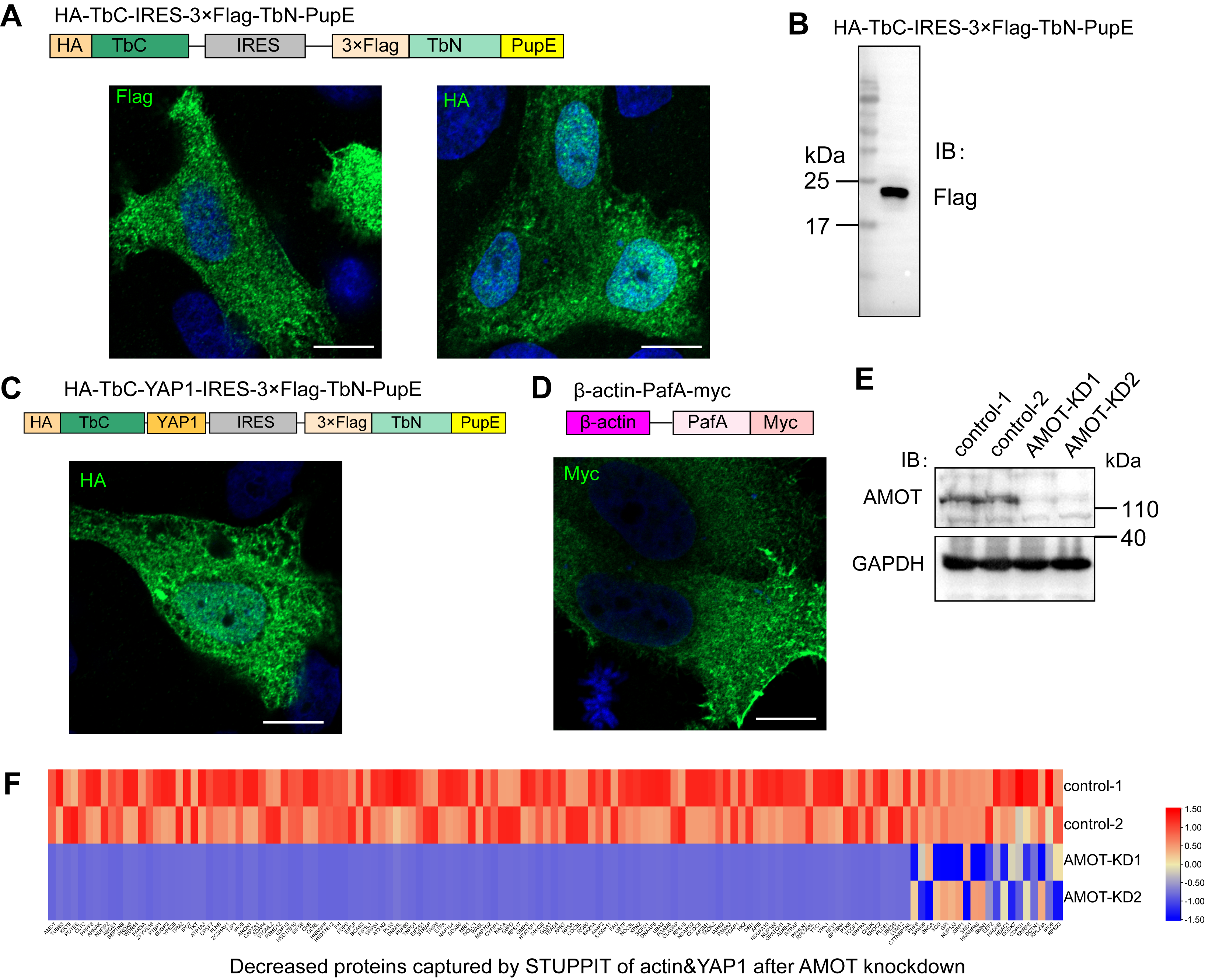

Supplement: S3 Fig — (A) Immunofluorescence staining of Flag and HA tag in HA-TbC-IRES-3 × Flag-TbN-PupE control vector transfected HeLa Cells. (B) Immunoblotting of Flag in HA-TbC-IRES-3 × Flag-TbN-PupE expressed HEK293T cells. (C) Immunofluorescence staining of HA tag in HA-TbC-YAP1-IRES-3 × Flag-TbN-PupE transfected HeLa cells. (D) Immunofluorescence staining of Myc tag in actin-PafA-Myc transfected HeLa Cells. Scale bars are all 10 μm. (E) Immunoblotting of AMOT and GAPDH in control and AMOT-KD cells. (F) Decreased proteins captured by STUPPIT of actin&YAP1 after AMOT knockdown compared to control after mass spectrometry using the all-in-plasmid method. Two replicates for each group were performed. Around 135 proteins among the approximately 700 proteins showed decreased abundance (with a fold change >1.5, calculated as control/AMOT-KD) upon AMOT knockdown. (TIF) [file pbio.3003227.s003.tif]

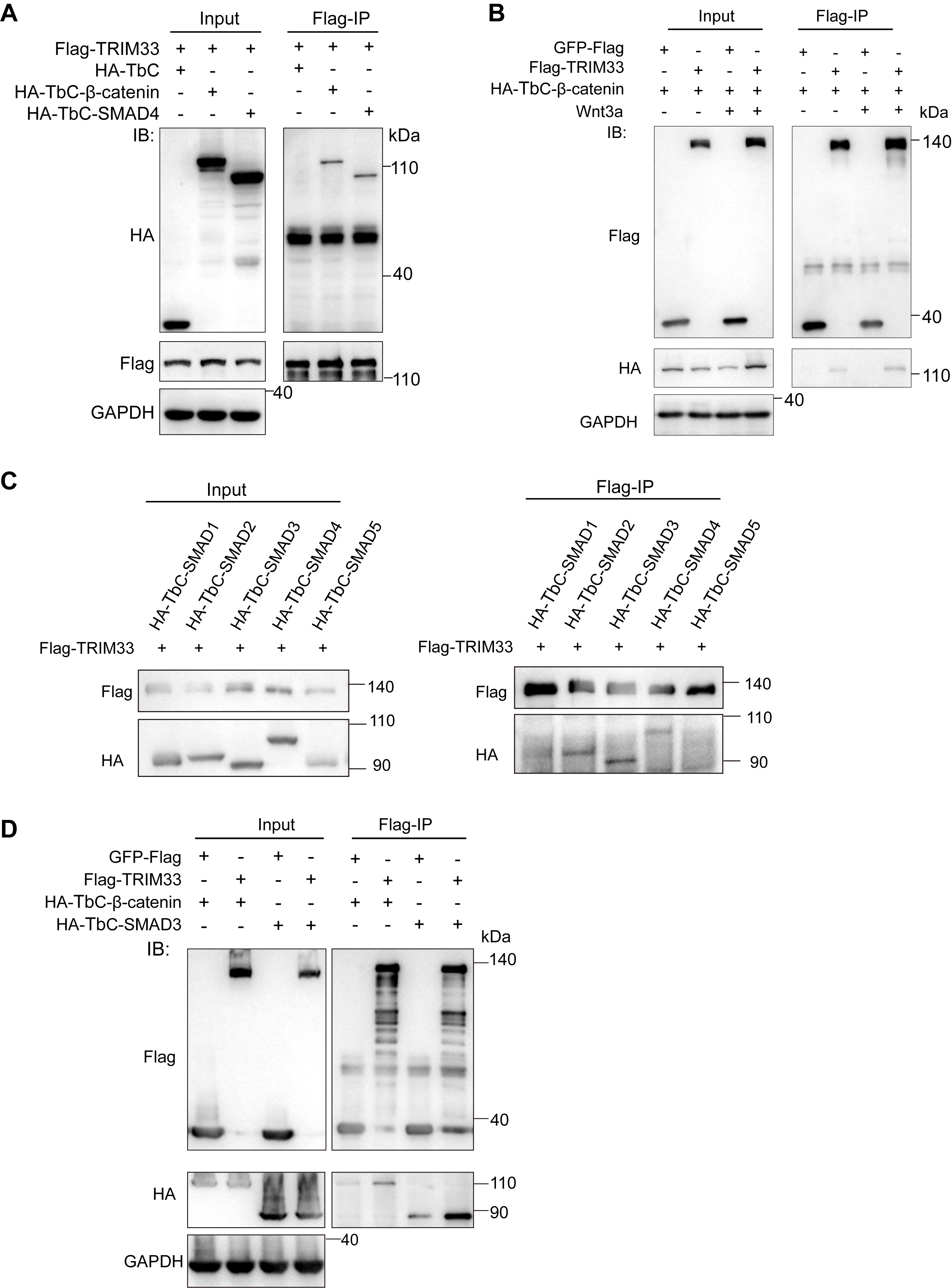

Supplement: S4 Fig — (A) Validation of the interaction between TRIM33 with β-catenin and SMAD4 captured by STUPPIT through immunoblotting. (B) The interaction between TRIM33 and β-catenin with or without Wnt3a stimulation by Flag-IP. (C) Validation of the interaction between TRIM33 with SMAD1, SMAD2, SMAD3, SMAD4, SMAD5 via immunoblotting following Flag-IP. (D) Validation of the interaction between TRIM33 with β-catenin and SMAD3 via immunoblotting following Flag-IP. (TIF) [file pbio.3003227.s004.tif]

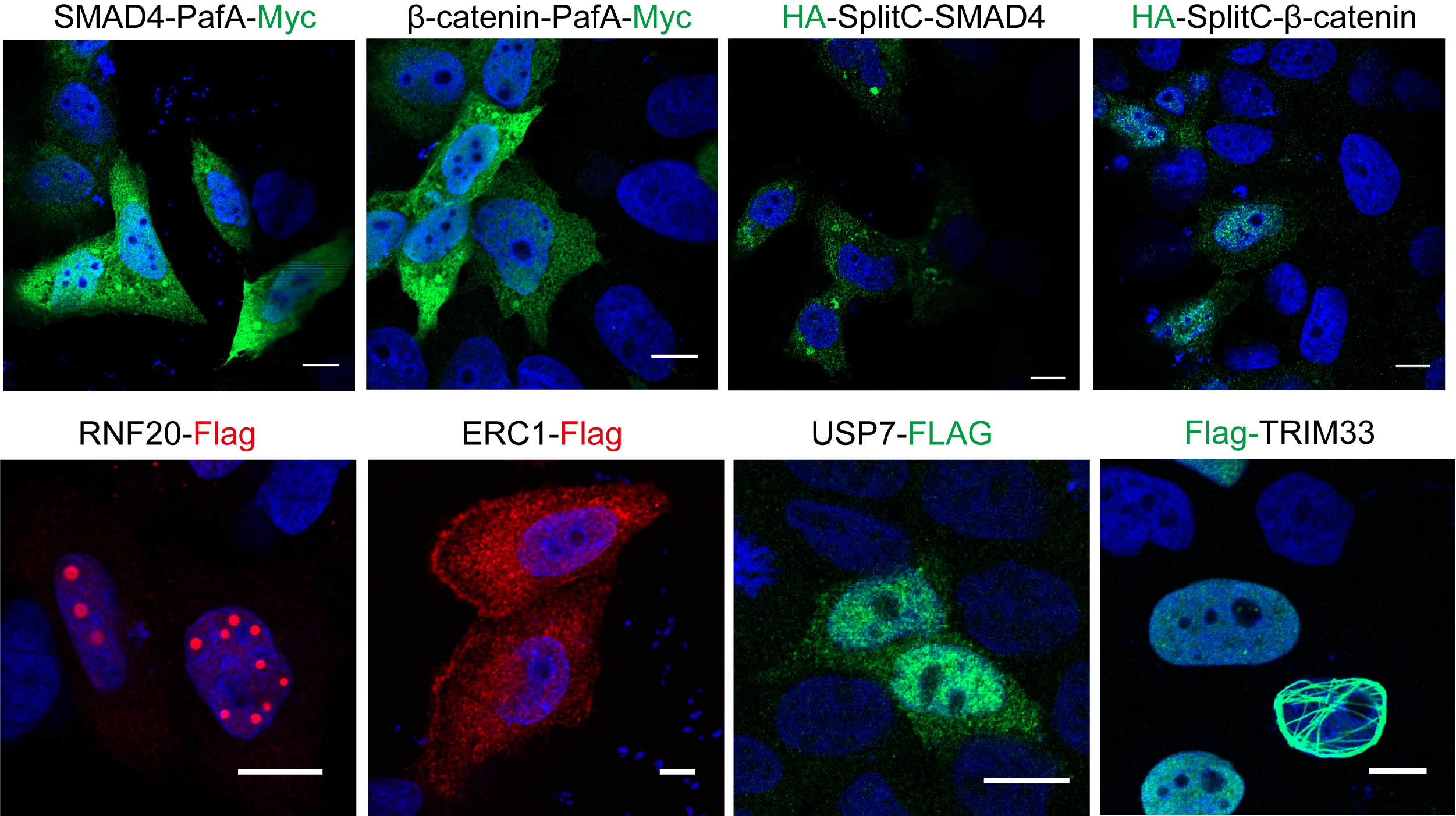

Supplement: S5 Fig — (TIF) [file pbio.3003227.s005.tif]

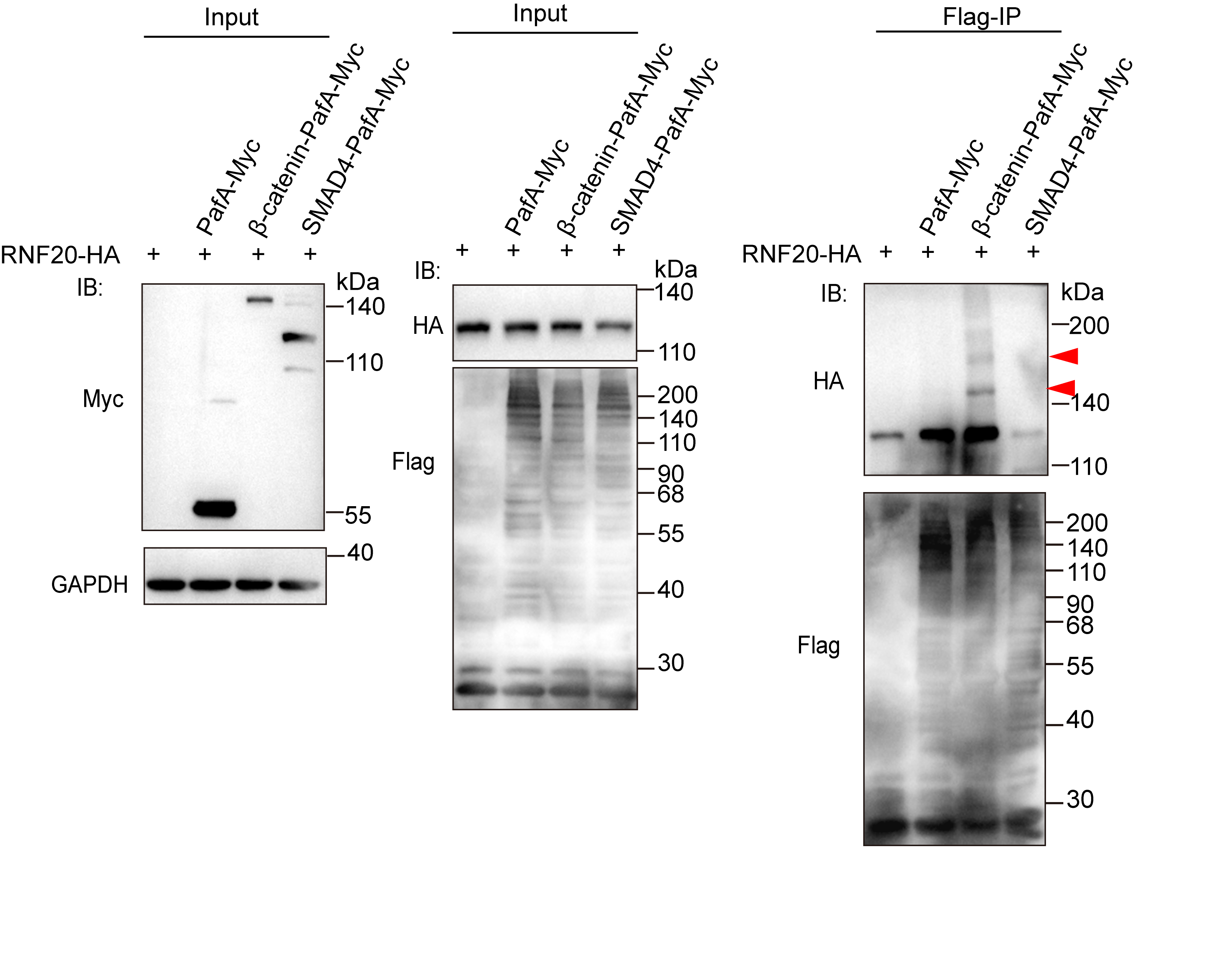

Supplement: S6 Fig — The immunoblots show that β-catenin-PafA-Myc but not SMAD4-PafA-Myc ligates 3 × Flag-TbN-PupE substrate to RNF20-HA. 3 × Flag-TbN-PupE stably expressed HEK293T cells were co-transfected with RNF20-HA and PafA-Myc, β-catenin-PafA-Myc or SMAD4-PafA-Myc, respectively. The red arrowheads indicate the molecular weight laddering of RNF20-HA after Flag-IP. (TIF) [file pbio.3003227.s006.tif]
