# Supplementary material for: STUPPIT is a proximity labeling tool for labeling intermediary proteins that bridge two non-interacting proteins
Source: PLoS Biol. 2025 Nov 24;23(11):e3003227. doi: 10.1371/journal.pbio.3003227 (PMC12668627; doi:10.1371/journal.pbio.3003227)
Supplement: S1 Raw Images — (PDF) [file pbio.3003227.s007.pdf]

Uncropped immunoblots in Figure 2

The sample sequence and annotations are consistent with those in the corresponding figure.

bands showed in the figure

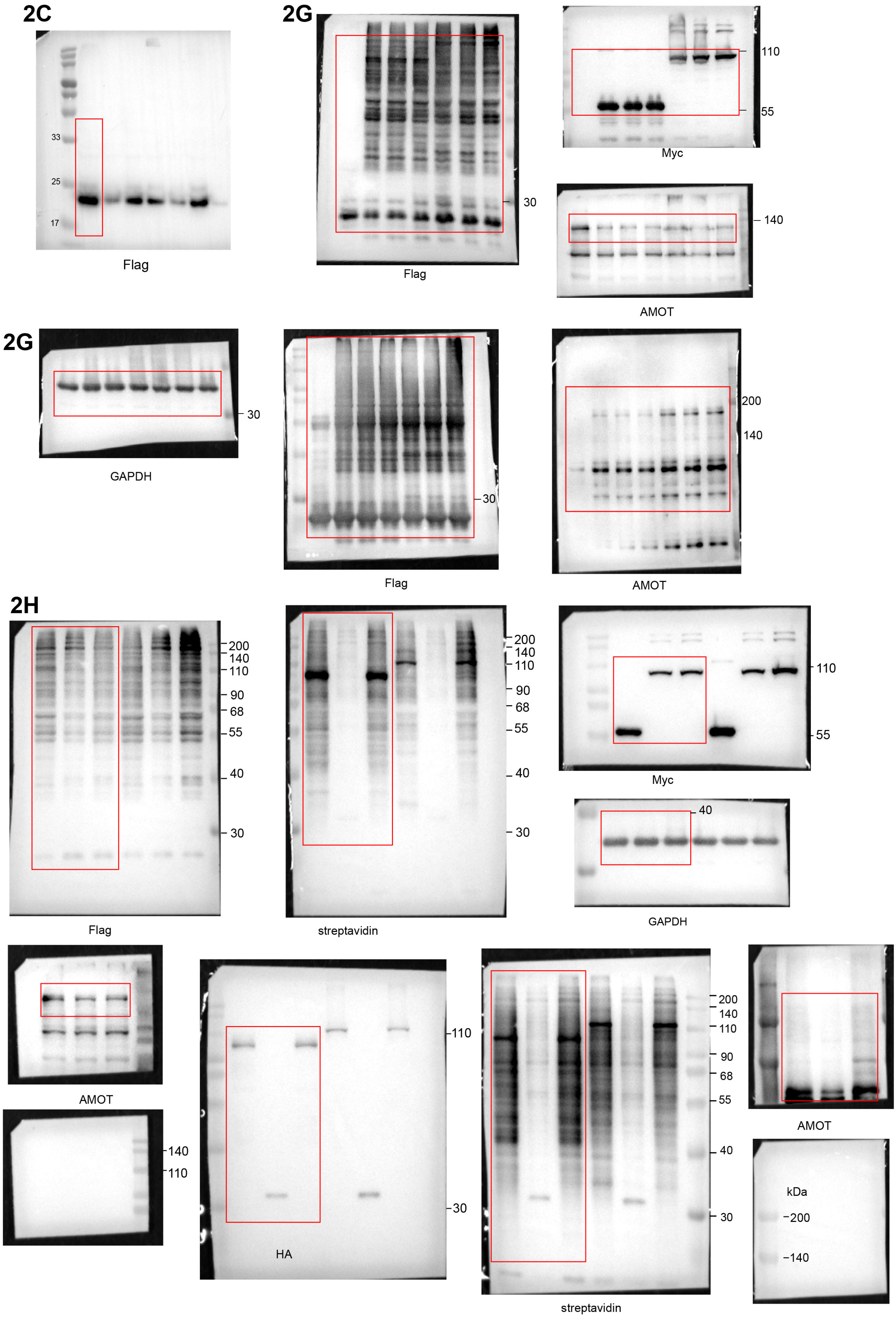

Uncropped immunoblots in Figure 3

The sample sequence and annotations are consistent with those in the corresponding figure.

bands showed in the figure

3D

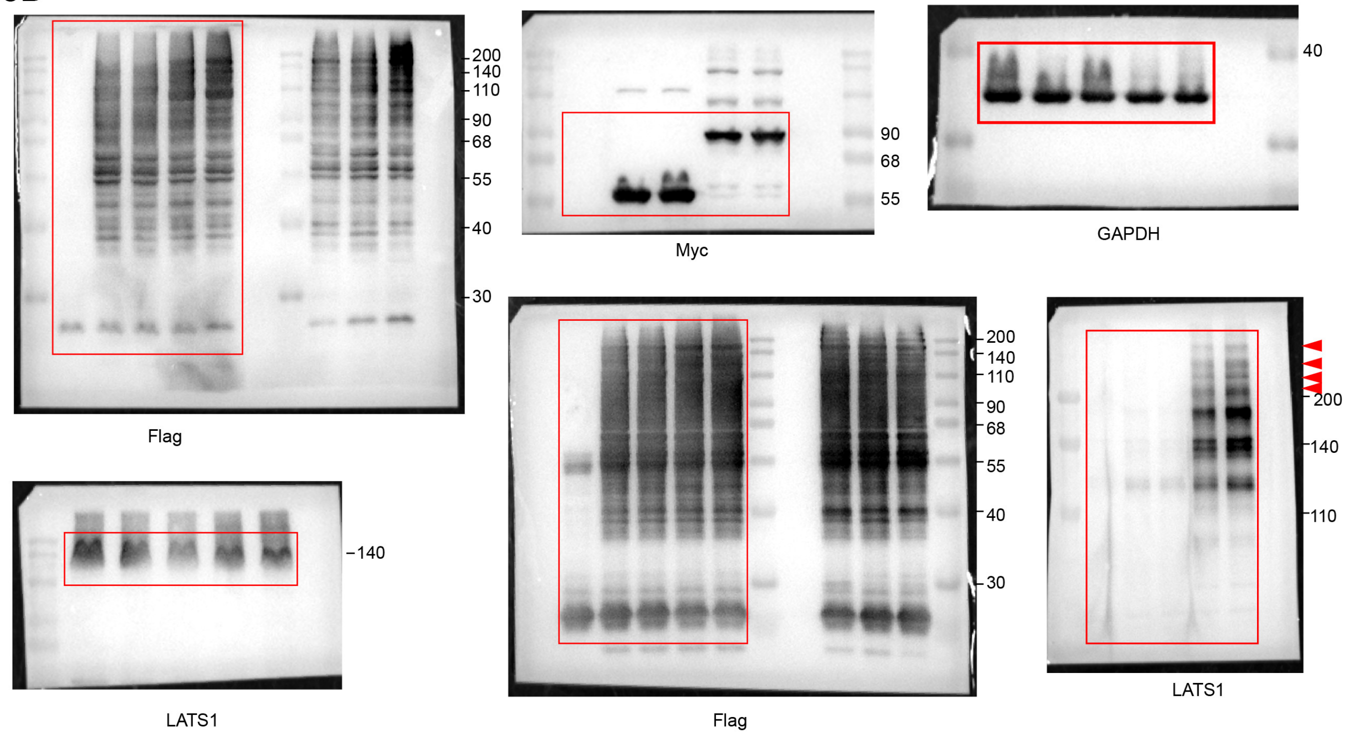

3E

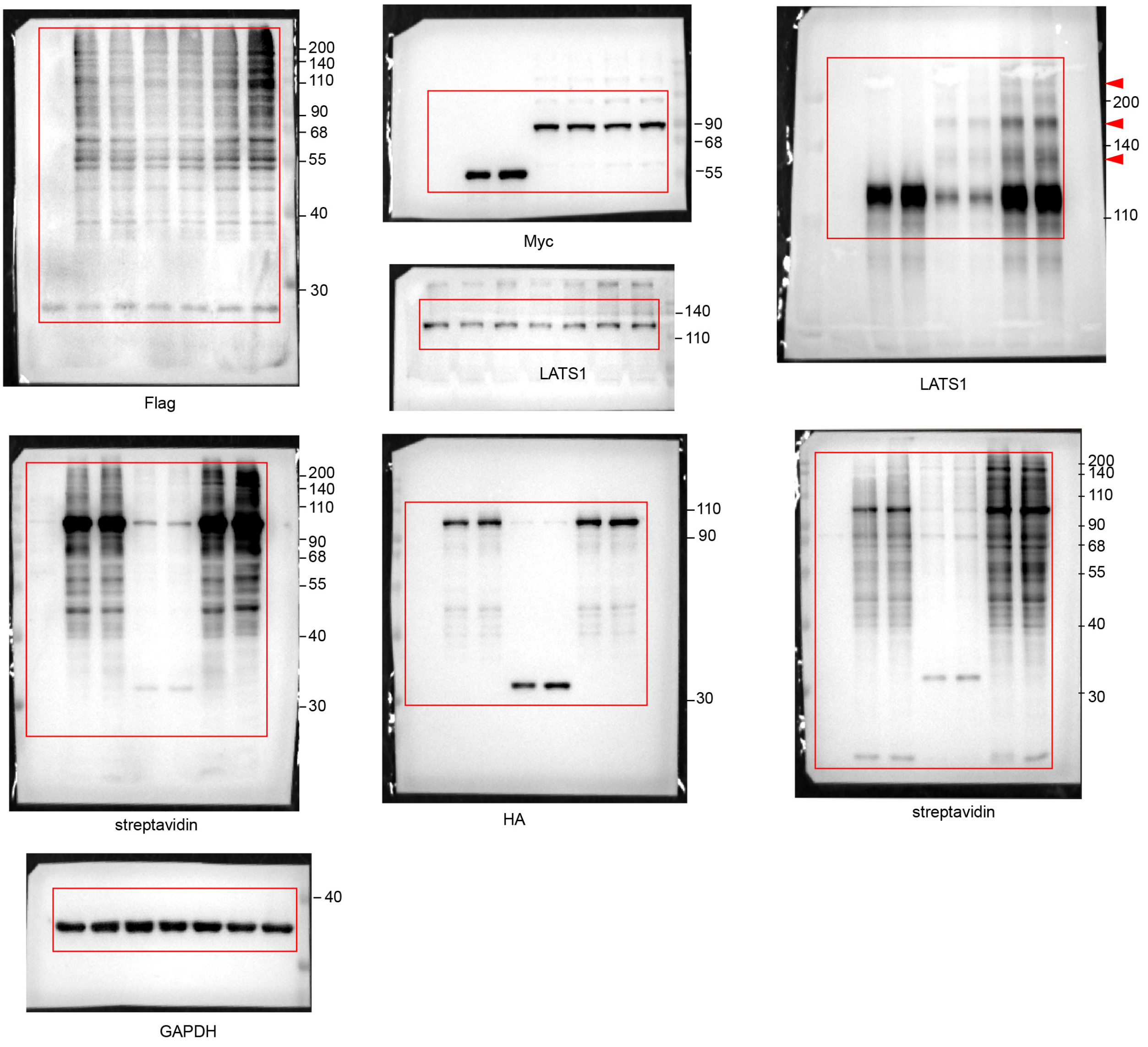

Uncropped immunoblots in Figure 4

The sample sequence and annotations are consistent with those in the corresponding figure.

bands showed in the figure

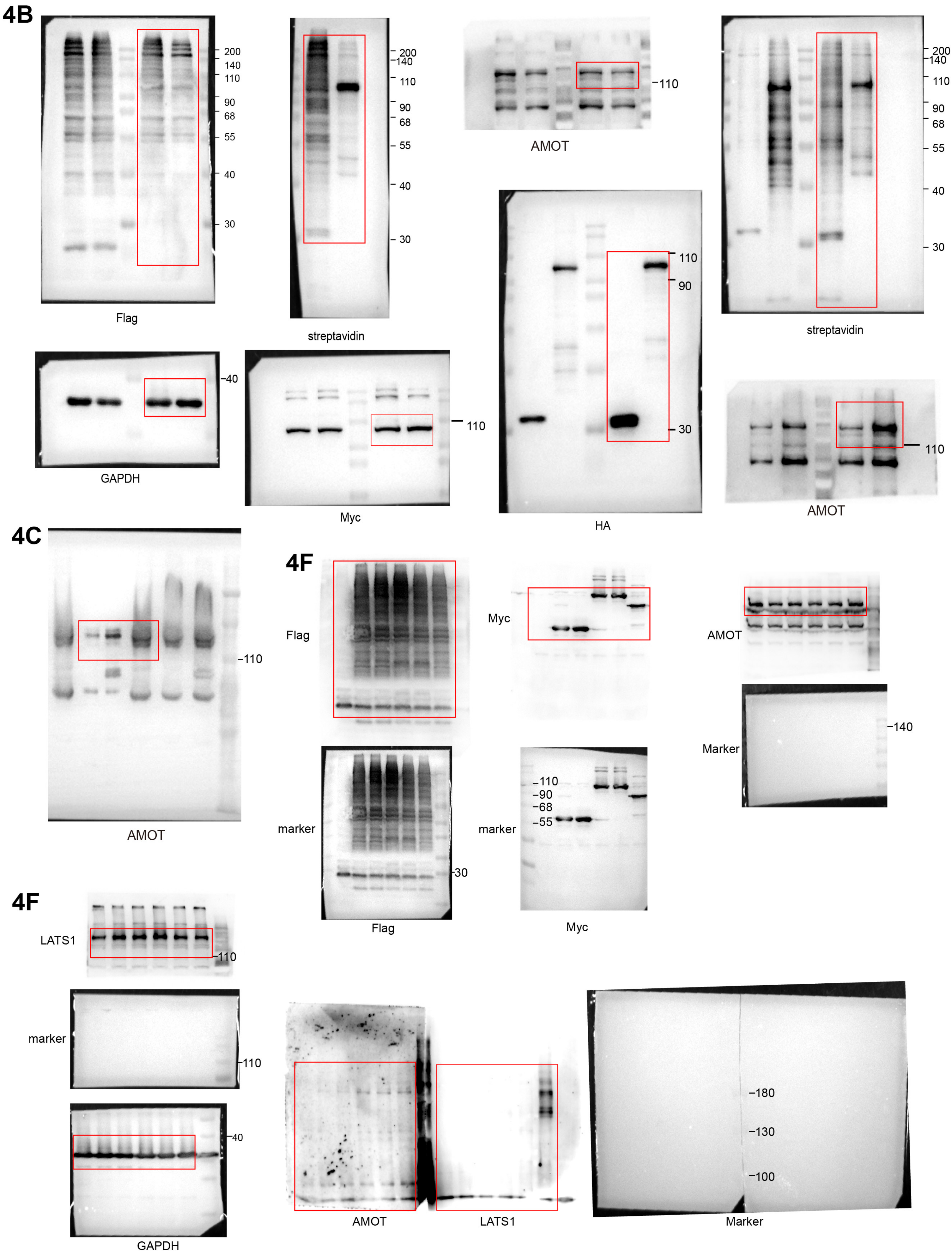

Uncropped immunoblots in Figure 5

The sample sequence and annotations are consistent with those in the corresponding figure.

bands showed in the figure

5C

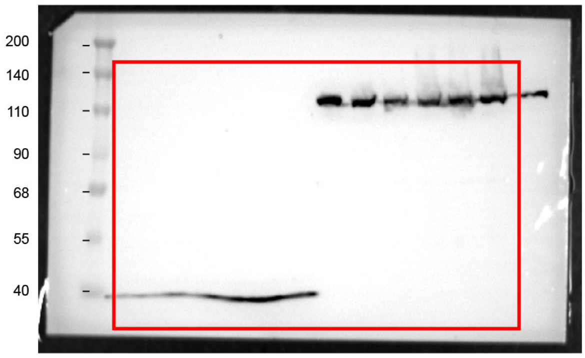

HA

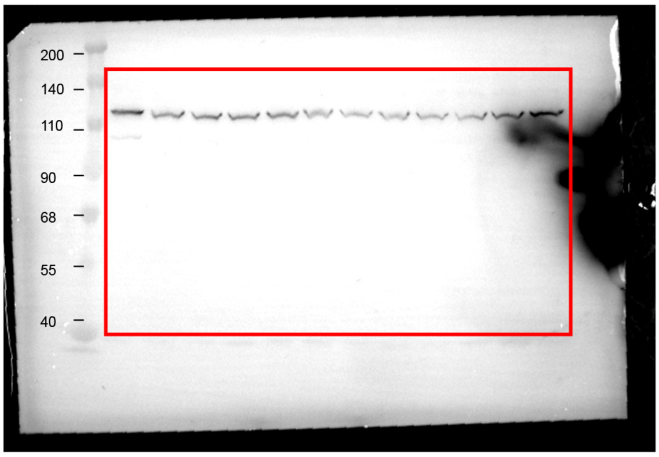

Myc

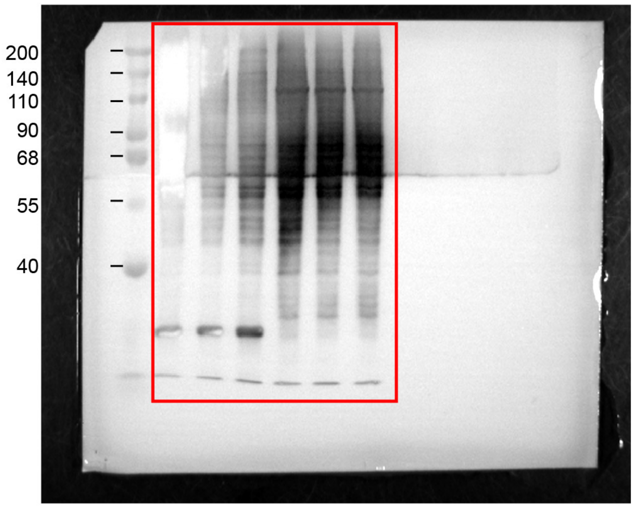

Streptavidin

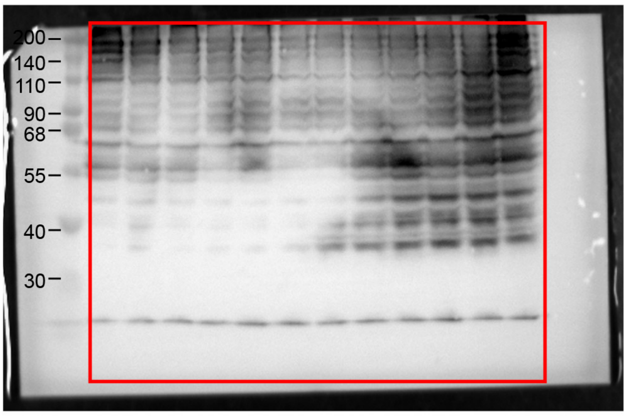

Flag

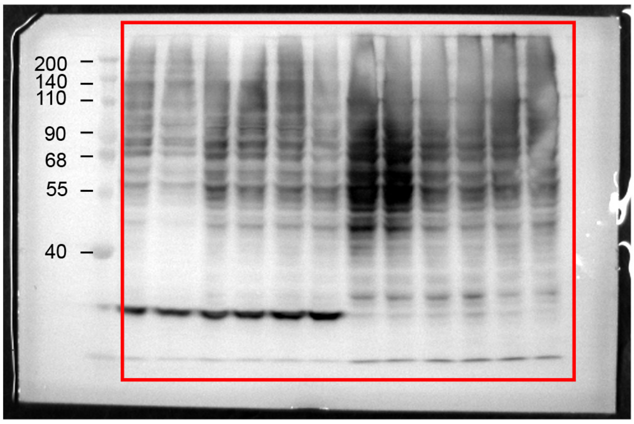

Streptavidin

Uncropped immunoblots in Figure 6

The sample sequence and annotations are consistent with those in the corresponding figure.

bands showed in the figure

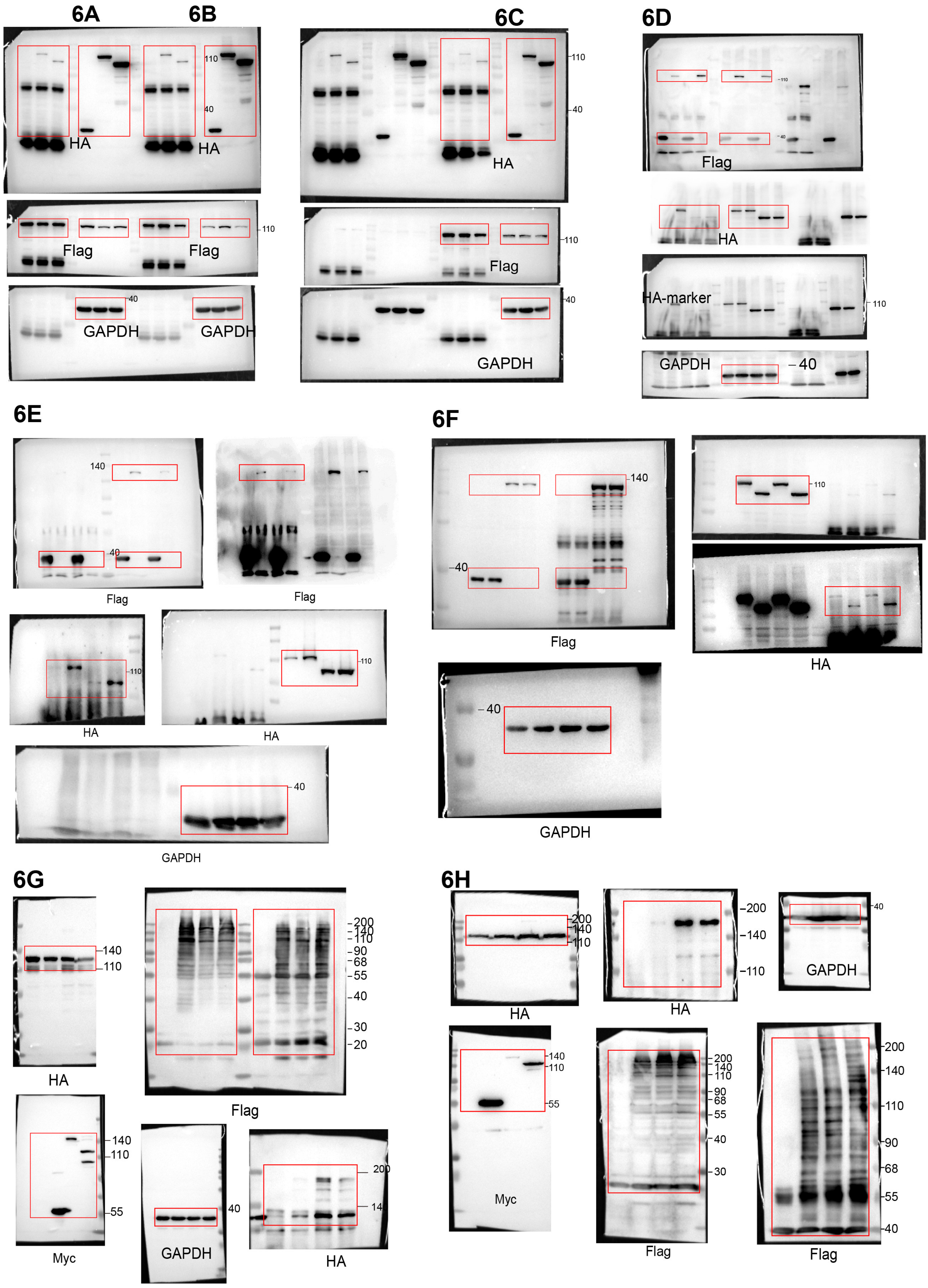

Uncropped immunoblots in Supplementary Figure 1

The sample sequence and annotations are consistent with those in the corresponding figures.

bands showed in the figure

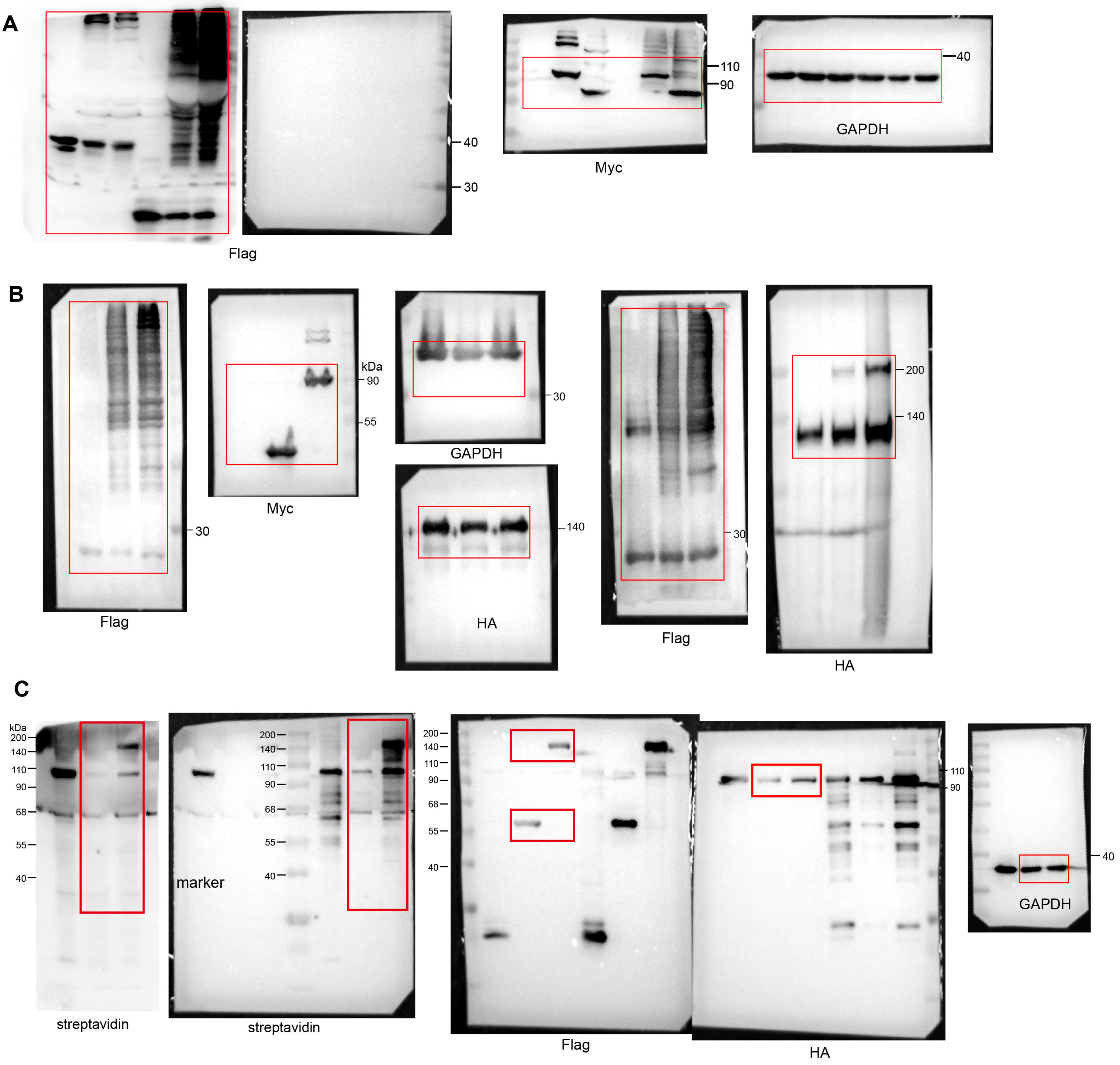

Uncropped immunoblots in Supplementary Figure 2

The sample sequence and annotations are consistent with those in the corresponding figure.

bands showed in the figure

C

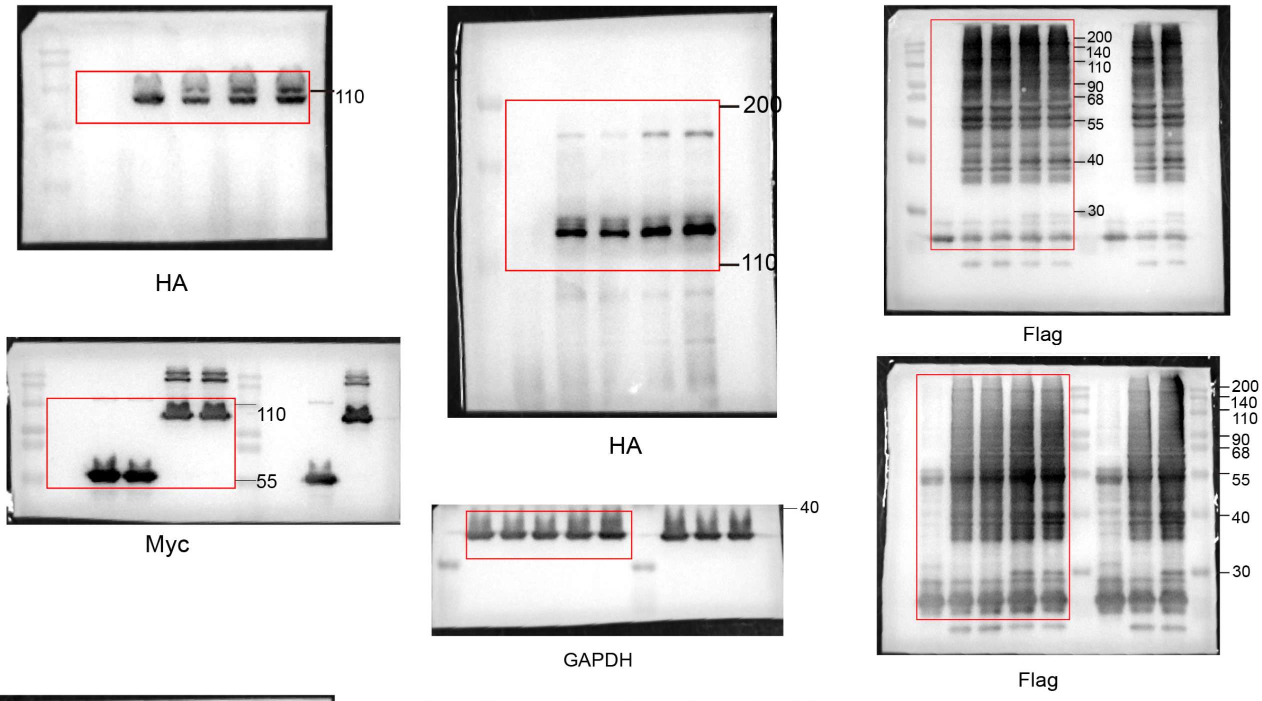

D

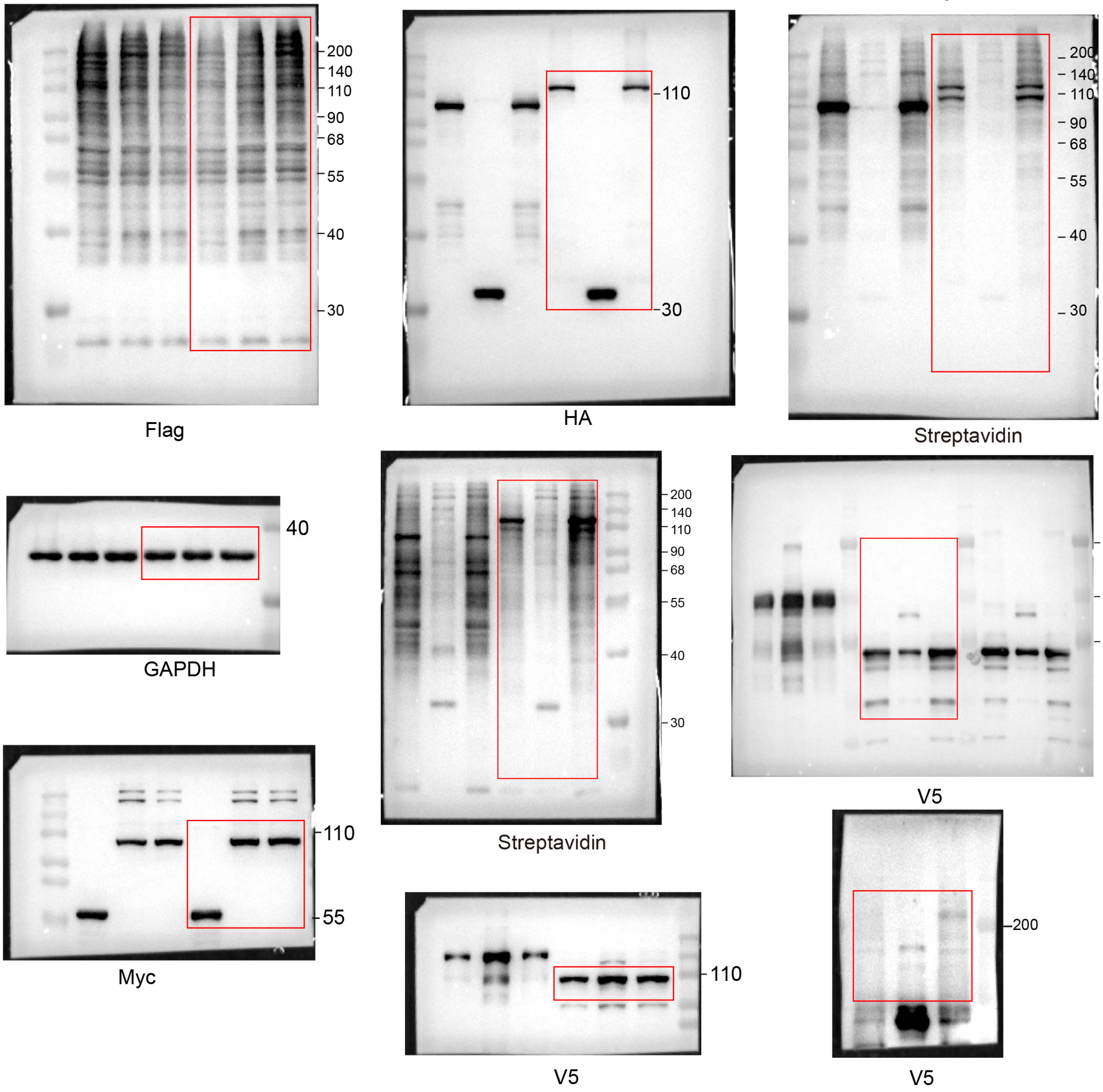

Uncropped immunoblots in Supplementary Figure 3

bands showed in the figure

The sample sequence and annotations are consistent with those in the corresponding figure.

B

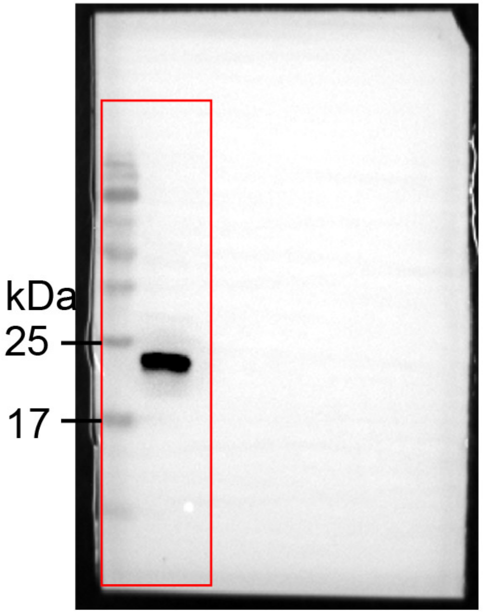

IB：Flag

E

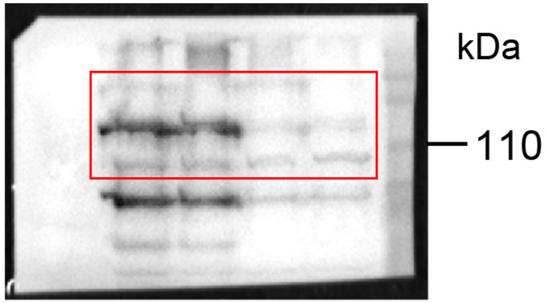

IB： AMOT

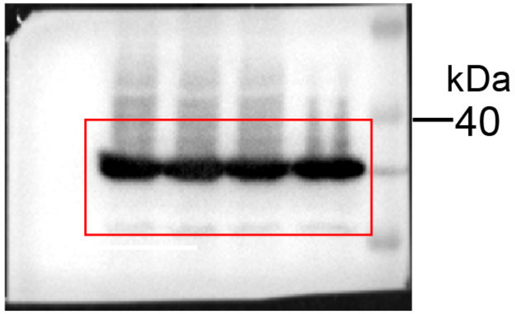

GAPDH

Uncropped immunoblots in Supplementary Figure 4

The sample sequence and annotations are consistent with those in the corresponding figure.

bands showed in the figure

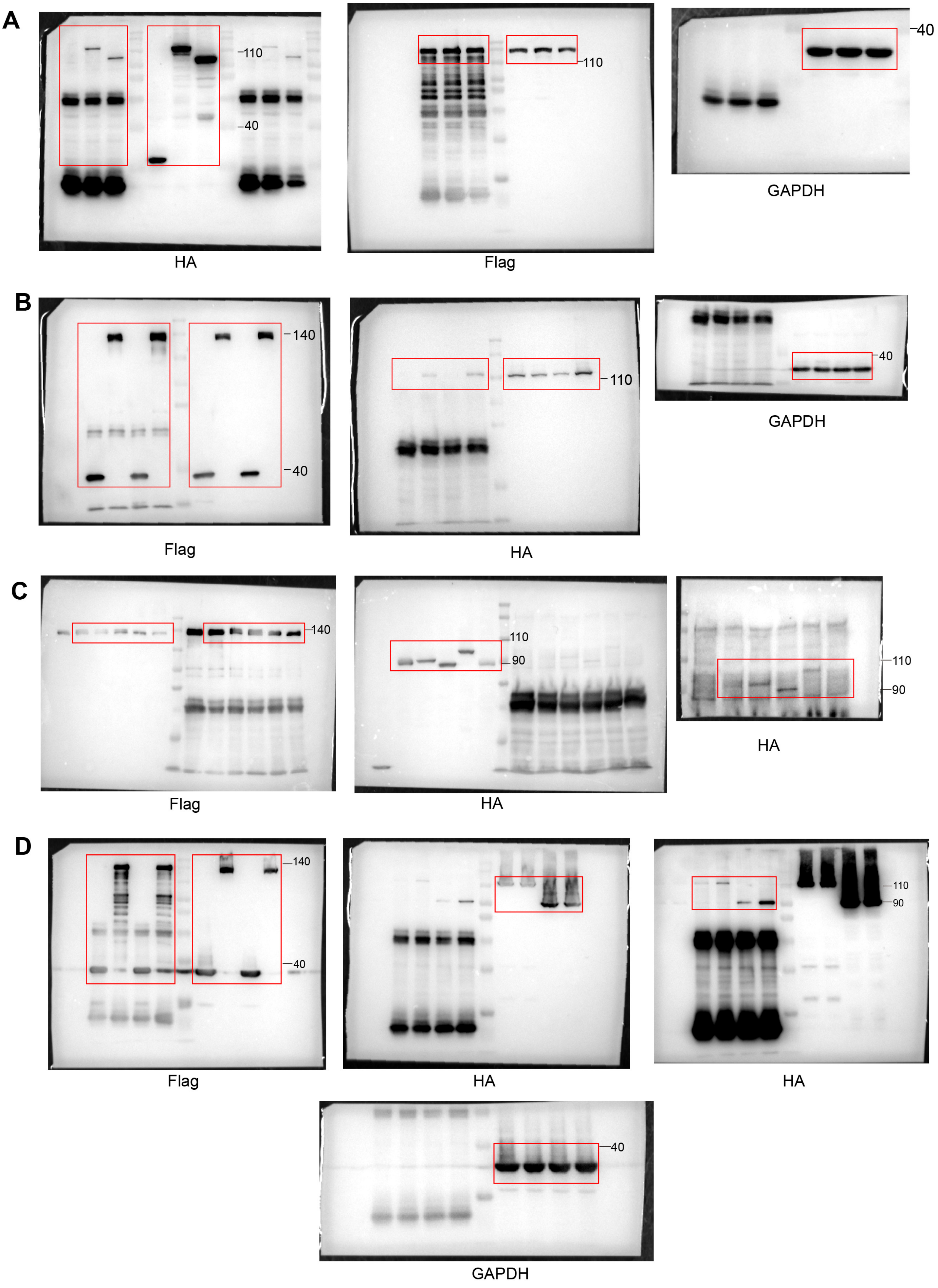

Uncropped immunoblots in Supplementary Figure 6

The sample sequence and annotations are consistent with those in the corresponding figure.

bands showed in the figure

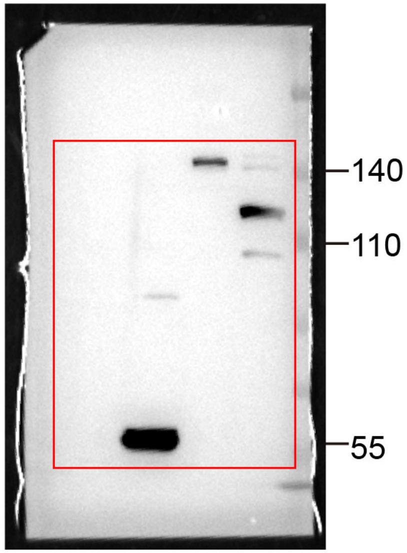

Myc

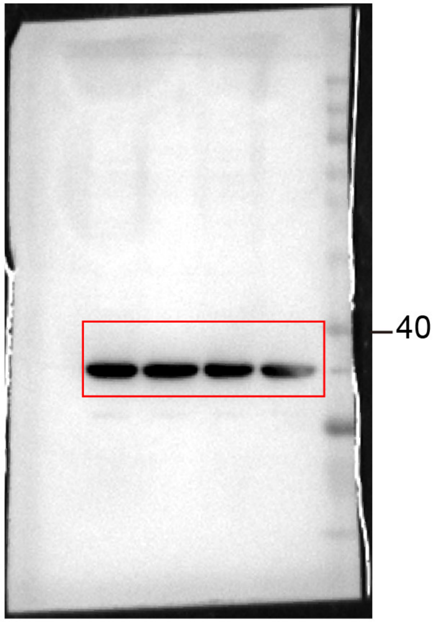

GAPDH

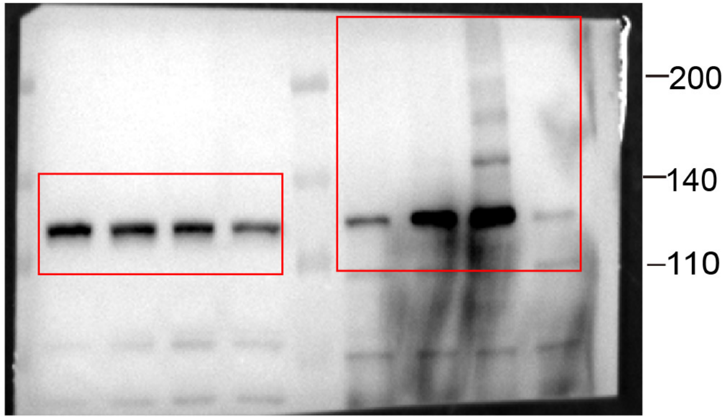

HA

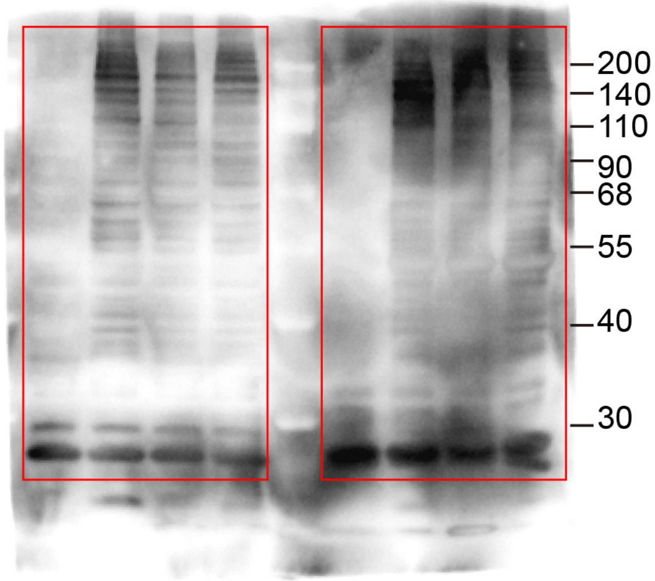

Flag
