# Supplementary material for: STUPPIT is a proximity labeling tool for labeling intermediary proteins that bridge two non-interacting proteins
Source: PLoS Biol. 2025 Nov 24;23(11):e3003227. doi: 10.1371/journal.pbio.3003227 (PMC12668627; doi:10.1371/journal.pbio.3003227)
Supplement: Attachment — Submitted filename: Response to Reviewers.pdf [file pbio.3003227.s008.pdf]

Response to Reviewers' comments

We have carefully considered the comments from the reviewers and have revised the manuscript accordingly. Below, we provide a point-by-point response to each of the concerns raised. All corresponding changes in the manuscript have been highlighted for the reviewers' convenience. We sincerely appreciate the valuable feedback, which has greatly improved the quality of our paper.

Reviewer #1: Sequential protein-protein interaction relays are critical for signal transduction, making the capture of these interactions essential. In this study, Xie et al. developed STUPPIT, a proximity labeling tool combining PUP-IT with split TurboID to identify network components when two distantly related proteins are known. The system is well-designed: the authors validated its feasibility using established PPI networks and applied it to map new components in the SMAD pathway. While validation of the newly identified intermediary protein between SMAD4 and catenin remains incomplete, this methodological study establishes a foundation for future investigations.

We thank you for your positive feedback and for recognizing the potential of our STUPPIT approach in studying sequential protein-protein interactions.

A few minor points need clarification:

1. What advantages does STUPPIT offer for identifying intermediate interactors between proteins A and B compared to performing proximity labeling on each protein individually and then cross-comparing results to find intermediate interacting proteins? Please elaborate on this comparison in the discussion.

We thank you for your comments and suggestion. We have now elaborated on this comparison in the discussion. "Performing separate proximity-labeling experiments on proteins A and B, followed by cross-matching of their biotinylated proteomes, can partially address this need. This strategy, nonetheless, is indirect and accompanied by additional drawbacks. For instance, to be detected as candidate intermediates, target proteins must not only be efficiently labeled by both baits but also pass two independent background filtering steps. Furthermore, it requires substantially higher sample input and incurs greater experimental costs. In contrast, STUPPIT offers a solution by enabling the specific direct labeling and enrichment of intermediary proteins between two non-interacting bait proteins. This unique capability positions STUPPIT as a valuable alternative tool for investigating signaling pathways, particularly those in which key components do not engage in direct physical interactions."

2. In Fig. 5E-H, proteins with identical tags should be presented in a single blot rather than separated by molecular weight. The current presentation obscures critical information about relative expression levels between control and test samples. In Figs. 3 & 4, the stronger biotin signals in control samples suggest that split TurboID exhibits high background when overexpressed in cells. Therefore, control Tb(C) and fused Tb(C) must be expressed at similar levels.

We sincerely thank you for this insightful observation. Regarding the original Fig. 5E–H, we note that most of the initial blots were processed by cutting during the original experiments. To address this, we have now repeated the Flag-IP assays and present samples with identical tags within the same lane of a

single blot wherever feasible, also for Fig 2-5. For instances where identical tags require longer exposure times and necessitate cutting (e.g., Fig. 6E and 6F), we have provided uncut blots with multiple exposure durations in the S1 uncropped raw images.

When co-expressing actin-PafA-Myc and TbN-PupE, we observed that the expression levels of larger fusion proteins (e.g., YAP-TbC) tend to be moderately lower than those of the control TbC. Despite this expression variability, AMOT was consistently enriched in the actin&YAP1 group compared to the control group. Additionally, following the recommendation from the split-TurboID study by the Alice Ting laboratory (Cho et al., 2020), we shortened the biotin treatment duration from the original 18 hours to 4 hours to minimize background labeling in the control split-TurboID. We have also repeated these experiments under strictly controlled conditions to ensure comparable expression levels across all samples (Figs. 2-4).

3. Why do RNF20, ERC1, TRIM33, and USP7 display different cellular localizations despite interacting with the same protein?

We thank you for this insightful question. Yes, the candidate proteins exhibit distinct cellular localization patterns, with RNF20 showing strong nucleolar localization. While for SMAD4 and β-catenin, by contrast, they are distributed in both the cytoplasm and nucleus. We propose that this localization difference may arise as follows: after biotinylation, some candidate proteins only interact transiently or weakly with SMAD4/β-catenin. This weak association allows them to dissociate from the bait proteins, after which they may interact with other proteins; such subsequent interactions could contribute to their distinct localization. Consistent with this hypothesis, our data demonstrate that ERC1, TRIM33, and USP7 exhibit stronger interactions with these bait proteins compared to RNF20 (Fig 6 & S5, S6 Fig).

4. PUPIT uniquely uses a peptide substrate for method development, but numerous split systems exist beyond split TurboID, including split APEX and split BioID. What is the rationale for selecting TurboID, and how broadly applicable is this system?

We appreciate this insightful question. Our selection of split-TurboID was based on multiple considerations. First, our lab already has extensive experience with TurboID and possesses the necessary plasmids, making split-TurboID a practical and well-established choice. Additionally, split-TurboID is widely adopted and frequently cited in the literature, underscoring its reliability. Another key factor is the recommendation from Min Zhuang's work that N-terminal tags fused to pupE should be kept short. Since split-TurboID is divided between residues 73 and 74, its N-terminal fragment consists only 73 amino acids, making it well-suited for fusion with pupE. This rationale is further supported by our experimental observation: when we fused the C-terminal fragment of split-TurboID (TurboC) to the N-terminus of PupE, the labeling efficiency mediated by pafA was significantly lower compared to that achieved with the short N-terminal fragment (TbN) (see figure below).

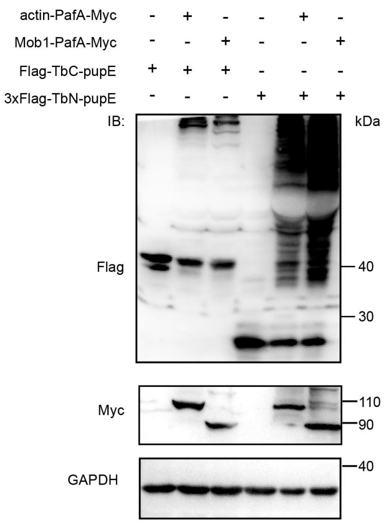

Theoretically, alternative split-protein systems-such as split-APEX, split-BioID, or split-GFP-could potentially substitute for split-TurboID, provided that one of their fragments is sufficiently small and compatible with fusion to PupE. Another critical consideration is that the split-X system must exhibit high specificity and low background noise when labeling interacting proteins. Notably, our system establishes a conceptual framework suggesting that combining different labeling tools or biochemical enzymes, including proximity labeling enzymes or even dCas9 for transcriptional level studies, could expand the potential for targeted labeling or functional analysis.

5. Fig 2A, 3A, should be "approach"

We sincerely thank you for identifying this oversight. The labels originally present in Fig. 2A and Fig. 3A have been revised to "approach" in the updated versions of the figures.

6. In Fig 3D, lanes (2) and (3) are labeled "IB:Myc" but should likely be "IB:HA."

We sincerely thank you for identifying this critical annotation error. To prevent similar oversights, we have also conducted a comprehensive review of all western blot annotations across the entire manuscript, ensuring the consistency and accuracy of target protein labels with the corresponding immunoblotting experiments.

7. Fig 5C: clarify the meaning of the numbers above the Western blot bands and provide additional detail in the figure legend.

We thank you for your suggestion and apologize for the unclear and incomplete description in the figure legend. Samples 1–6 correspond to 6 replicate experiments from the SMAD4&TbC control group, each conducted in 6 cm dishes. Samples 7–12 correspond to 6 replicate experiments from the SMAD4&β-catenin experimental group, also performed in 6 cm dishes. To ensure sufficient protein enrichment for subsequent analyses, the 6 replicate samples in each group were pooled into 3 biological replicates. This detail has now been added to the relevant figure legend for clarity.

Reviewer #2: In this manuscript Xie L. et al. present an interesting method to study protein-protein interactions that aims at addressing the question: if considering protein A and protein C that are not physically directly interacting with one another, is it possible to identify proteins that interact with C after a prior interaction with A. Such potential proteins are dubbed "intermediary proteins" and may be especially relevant in signalling cascades.

The method is based on a clever setup that combines the PUP-IT and split-turboID techniques in an approach termed STUPPIT. In PUP-IT, the PafA protein ligase is expressed in mammalian cells in presence of its protein substrate Pup(E), PafA then non-specifically conjugates Pup(E) on lysine residues of any nearby protein. In split-turboID, two complementary fragments of the protein biotin ligase turboID are fused to two interacting proteins that trigger re-assembly of the active turboID enzyme, leading to non-specific biotinylation of neighbouring proteins.

In STUPPIT, a first bait protein (A) is fused with PafA: Prey A-PafA. In contrast to PUP-IT, the subtrate for PafA is however a fusion protein made of one fragment of split-turboID fused with pup(E): Tb(N)-pup(E). Then a second bait protein (C) is fused with the complementary split-turboID fragment: Tb(C)-Prey C. The idea is that if a protein interacts with both Prey A and Prey C (be it simultaneously or sequentially), it will first be conjugated with Tb(N)-pup(E) because of the interaction with Prey A and then activate turboID-mediated biotinylation due to the interaction with Prey C and re-assembly of the Tb(C) and Tb(N) fragments.

The manuscript is generally clear and the experiments well-conceived. The method is potentially interesting but to my opinion some more control experiments are needed to demonstrate that it is really filling a missing spot in the toolbox of methods available for the study of protein-protein interactions. In addition the description of the method is several times ambiguous as to its capacity to identify intermediary proteins or solely proteins that are parts of a complex that contains C in addition to a protein that previously interacted with A (both are not the same!)

One easy additional experiment that the authors should perform to demonstrate the utility of their method is performing split-turboID experiments with all the baits A and C used in this study. The authors write that split-turboID and related-techniques are limited to proteins that physically interact directly, this was to my knowledge often claimed but never really demonstrated: physically what matters is if the linkers allows the re-assembly of the two halves within a complex, if this automatically correlates with the necessity to be fused to two directly interacting proteins does not seem to be something to take for granted. If the authors do not observe biotinylation for all their baits, then they would show that STUPPIT brings something more than split-turboID.

We sincerely appreciate your positive evaluation of our work and the insightful questions that help refine the rigor and clarity of STUPPIT's utility.

As originally established by Alice Ting and colleagues (Cho et al., 2020), the split-TurboID system can be split at multiple sites (e.g., 256/257, 78/79, 73/74). Among these, the 73/74 split-TurboID exhibited minimal background signaling under the no-interaction control condition (co-expression of TbN and TbC, no rapamycin), while strong streptavidin signals were detected when the fragments were brought together via rapamycin-induced strong interactions between FKBP and FRB (see their original figures below Fig. 1D). Notably, even the TbN&TbC control condition still showed weak background signals, as evident in both their immunostaining (Fig. 2D) and immunoblotting (Fig. 3E) results. It is a common and inherently difficult-to-avoid phenomenon for TurboID, APEX, and other split-protein systems (e.g.,

split-GFP) as well. Additionally, their Fig. 3E clearly indicated that ligase self-biotinylation and TurboID enzymatic activity were far more robust—with stronger signals—in the rapamycin-induced strong interaction group compared to the weak interaction control group (self-biotinylation is marked by "*" in their blot). They concluded that splitting TurboID at residues 73/74 generates two inactive fragments, which can recombine into an active enzyme when driven by a specific protein–protein interaction. This is exactly why we choose the split-TurboID (73/74) for the STUPPIT.

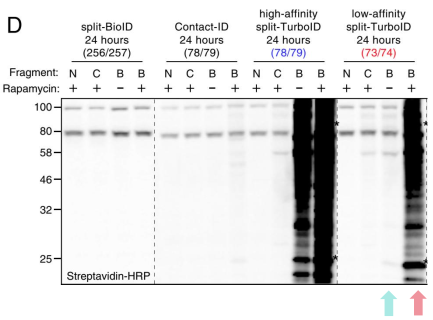

Fig 1D

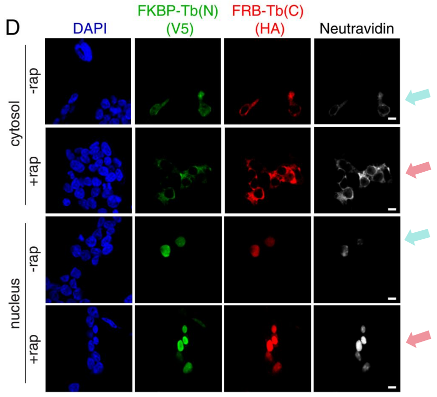

Fig 2D

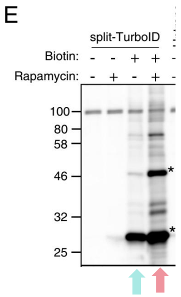

Fig 3E

Images obtained from split-TurboID paper (Cho KF, et al., PNAS, 2020)

* Green arrow indicates no-interaction control condition (TbN&TbC 73/74, no rapamycin), while red arrow indicates rapamycin-induced strong interactions between FKBP and FRB (TbN&TbC 73/74, with rapamycin).

To experimentally verify this in our contex, we further generated two constructs AMOT-Flag-TbN and actin-Flag-TbN. Using these, we assessed the ligase self-labeling and split-TurboID (split at 73/74) activity under two conditions: AMOT-TbN co-expressed with TbC-YAP1, and actin-TbN co-expressed with TbC-YAP1. Consistent with the conclusions from (Cho et al., 2020) in their Fig. 3E, we observed that both the ligase self-biotinylation and TurboID enzymatic activity were far more robust—with significantly stronger signals—in the AMOT-TbN&TbC-YAP1 group than in the actin-TbN&TbC-YAP1 group. This result aligns with the known stronger interaction between AMOT and YAP1 (compared to actin and YAP1), further validating that split-TurboID (73/74) activity directly correlates with the strength of the interaction driving fragment proximity.

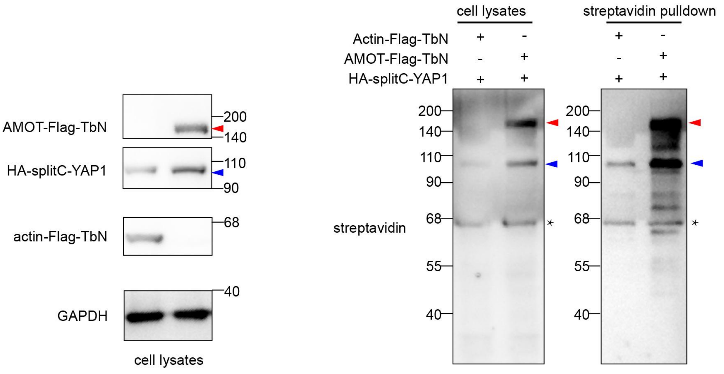

Arrowheads indicates ligase self labelling, while * indicates non-specific bands.

In implementing STUPPIT, we adopted several strategies to enhance specificity. Based on recommendations from the Ting lab (Cho et al., 2020), we shortened the biotin incubation time to 4 hours to reduce background labeling, as opposed to the 18 hours used in our earlier experiments. To further minimize background in the STUPPIT system, we intentionally maintained only one variable component the TbN-PupE fusion while keeping all other elements consistent, including PafA expression levels and biotin concentration. This design minimizes nonspecific biotinylation and improves the signal-to-noise ratio critical for reliably identifying intermediary proteins.

Additionally, as suggested later, we also included an additional control group in which PafA was omitted from the system while retaining TbC-PreyC. Using cells stably expressing the substrate TbN-PupE, we performed STUPPIT with the actin&YAP1 bait pair and a control group expressing only TbC-YAP1 with no PafA, with two biological replicates. Consistent with our initial MS results where actin-PafA&TbC via the all-in-plasmid method served as the control (now Figure 4), AMOT was enriched in the actin&YAP condition relative to the YAP-only control. Notably, LATS1, a well-documented direct interactor of YAP, was not enriched in the actin&YAP STUPPIT sample, but was detected in the MOB1&YAP condition. These findings support the conclusion that STUPPIT selectively identifies intermediary proteins between two prey proteins, rather than all proteins associated with prey protein C.

Here are more detailed comments to the authors:

1) Lines 124/125: "this method designed to tag the intermediary connectors of two non-interacting proteins." Lines 147/149: "Subsequent steps involve utilizing streptavidin affinity immunoprecipitation and mass spectrometry proteomics to capture and identify the intermediary proteins"

To be precise, the method will tag proteins that are proximal to A (with pup(E)), one or some of them (protein(s) X) will also interact with C and those proteins that are proximal to X/C will be tagged with biotin, X will be part of it but among many others. Since the detection is solely based on biotin, the method cannot be specific to "intermediary proteins" (= those that got biotin and pup(E)).

This makes the experiment depicted on Fig. 2 more suggestive than conclusive. Fig. 2C suggests that AMOT will be the protein that undergoes first conjugation with Tb(N)-pup(E) and then activate Tb(C)-YAP1. While this would be logical and the data suggest it is what happens, this has not been fully demonstrated. Indeed another Protein X may be conjugated by Tb(N)-pup(E), then activate Tb(C)-YAP1 and lead to biotinylation of AMOT because it interacts with YAP1. The fact the AMOT runs slower on an SDS-PAGE upon transfection of PafA-β-actin suggests that it got conjugated with Tb(N)-pup(E) but this was not demonstrated.
If the authors really want to show their model holds, they may perform one of these control experiments:
- Immunoprecipitation with an anti-Flag antibody of the samples of Fig. 2F and immunoblot with anti-AMOT antibody: this will show that the size shift is indeed due to conjugation of Tb(N)-pup(E)
- STUPPIT like in Fig. 2E-G in cells in which AMOT was knocked down: if biotinylation and MS data are significantly changed this will show that AMOT was indeed the main intermediary protein
Without at least one of these two controls, if AMOT was identified because it interacted with actin prior it interacted with YAP1 is only supported by the current literature.

If done for approach I, the suggested control experiments of course do not need to be performed for approach II.

We appreciated your suggestions and we noticed that this is indeed very important for our work. We performed both these two control experiments.

- Specifically, we conducted the immunoprecipitation (IP) using anti-Flag beads to validate whether actin-PafA can label the Flag-TbN-PupE substrate onto the intermediate protein AMOT. If this is the case, AMOT should be detectable following Flag-IP and exhibit molecular weight laddering as described. As expected, both endogenous and exogenous AMOT were successfully enriched following anti-Flag enrichment and exhibited molecular weight laddering detected by anti-AMOT antibody, comparing to the no-transfection or the pafA-myc transfection control (Fig 2G and S1B Fig).

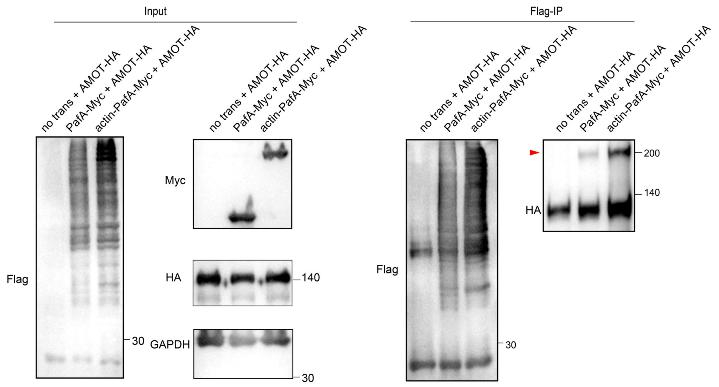

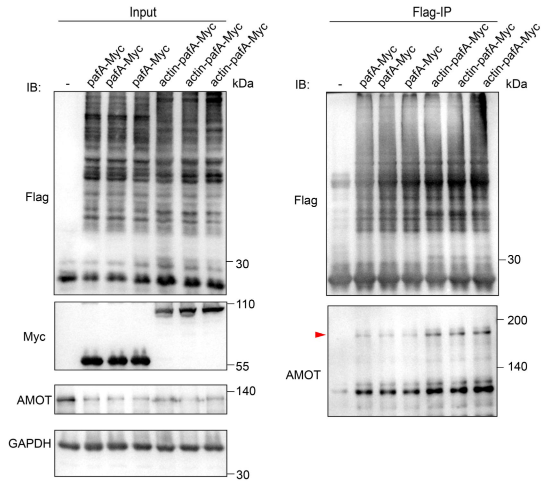

We also performed the second experiment you suggested. We conducted STUPPIT analysis of the actin&YAP1 bait pair in both control stable cell lines and AMOT-knockdown stable cell lines, using the all-in-plasmid method, followed by mass spectrometry (with two biological replicates). Among the approximately 700 proteins identified in the analysis, around 135 proteins showed decreased abundance (with a fold change > 1.5, calculated as control/AMOT-KD) upon AMOT knockdown.

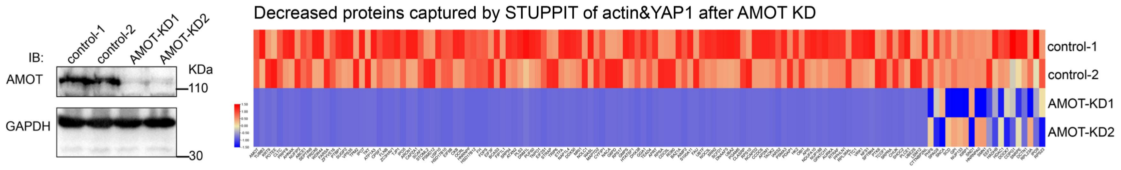

2) For all the western blots presented in this manuscript: inputs are missing a normalization control (total protein stain or housekeeping control)

We sincerely thank you for this critical suggestion, as normalization controls are essential for ensuring the reliability of Western blot data. We have now repeated the majority of the experiments in this paper and included GAPDH as a housekeeping control for normalization.

3) Approach II is depicted on Fig. 3 and is in principle interesting, however the data on Fig. 3D are confusing:

** FLAG-blot, lanes 2 & 3: why is there no Tb(N)-pupE to see (it should be stably expressed)*

We sincerely thank you for raising this point. In the original Fig. 3D, the stably expressed Tb(N)-PupE protein was indeed present in lanes 2 and 3, but its band appeared relatively faint, likely due to the stronger signal intensity from adjacent bands, under the chose exposure condition. A longer exposure of the same blot clearly confirms the presence of Tb(N)-PupE band in these lanes. Additionally, considering that new figures (e.g., revised Fig. 2 and 3) include updated, more comprehensive experiments with consistent and clear detection of stably expressed Tb(N)-PupE, we have decided to remove the original Fig. 3D to avoid potential confusion and streamline the presentation.

** Streptavidin, lane 2: considerable biotinylation is observed with two fragments that are just floating around (Tb(C) and Tb(N)-pupE): why? Tb(C) & Tb(N) seems to self-assemble in the absence of actin-pafA. If true then the right negative control for STUPPIT is without pafA.*

We thank you for this important observation. The background biotinylation observed in lane 2 is likely resulted from the extended biotin incubation time used initially. In line with the protocol recommended by the Alice Ting lab, we evaluated shorter labeling durations and confirmed that background signal increases substantially with longer incubation (e.g., 18 hours, as originally used) compared to a 4-hour treatment. Accordingly, we have updated all the streptavidin pulldown experiments to use the 4-hour biotin labeling condition.

We fully agree that omitting PafA serves as an essential negative control to assess any autonomous labeling of YAP-interacting proteins. To address this, we included an additional control in which PafA is omitted while YAP-TbC was retained, followed by mass spectrometry analysis. As summarized in our response to Question 6—and consistent with the initial MS results from Approach I (Figure 2)—AMOT was enriched in the actin&YAP condition compared to the YAP-TbC (PafA-omitted) control. Importantly, LATS1, a known direct interactor of YAP, was not enriched under this STUPPIT condition, but was clearly detected in the MOB1&YAP positive control. These results reinforce that STUPPIT selectively captures intermediary proteins rather than all YAP-associated interactors that may be captured by background labeling, thereby underscoring the specificity of the method.

** Myc blots do not seem to make much sense for lanes (2) and (3): why should the size change, why should there be a signal at all when actin-pafA is not expressed?*

Thank you for catching this critical inconsistency, which arose from an annotation error in the original figure. The labels for lanes (2) and (3) were incorrectly marked as "Myc" and should be "HA" in the

original figure. We apologize for this oversight.

* *In any case, please include a full blot for Myc with all samples (like for Flag & Strep)*

Thank you for this suggestion. We have now repeated all the original experiments and included a full Myc blot containing all samples, as requested.

4) The data presented on Fig. 3d and 3f do not correlate.

We thank you for this helpful suggestion. Additionally, given that the revised Figure 2 and Figure 3 already include data consistent with the original Figure 3D, we have removed the original Figure 3D from the revised manuscript to avoid redundancy and clarify the data presentation.

5) Figure 4D: first blot should be IB:Flag (I guess) not IB: Streptavidin

We thank you for pointing out this inconsistency. We have carefully rechecked all Western blot labels and made the corrections in the revised figure.

6) Fig. 4C: If the data presented on Fig. 3D is right, the suitable control seems to be without Pap: how much reassembly would you already have with Tb(C) floating around?

Thanks for your question. We wish to keep only one variant in the STUPPIT system, so we used actin-pafA&TbC&TbN-pupE as a control for actin-pafA&YAP1-TbC&TbN-pupE STUPPIT. As you suggested, we also tried to keep YAP-TbC in the STUPPIT system while taking out actin-pafA to exclude the background caused by YAP-TbC&TbN-pupE. We performed the STUPPIT of actin&YAP1, with YAP1-TbC (taking out of PafA) as control (two repeats). Consistent to the first MS results, AMOT was also enriched in the intermediary proteins in actin&YAP vs YAP control, but LATS1, which interacts with YAP, was not enriched in actin&YAP group after STUPPIT. We also conducted MOB1&YAP1 vs YAP1 without PafA. The MS proteomics data were different compared to actin&YAP1 group. The intermediary protein of LATS1 was enriched after STUPPT of MOB1&YAP1 as well. We also noticed AMOT was enriched in MOB1A&YAP1 but not LATS1. Mana-Capelli, S., & McCollum, D. reported that AMOT collaborates with MOB1 to enhance LATS1/2 autophosphorylation (Mana-Capelli and McCollum, 2018). Interestingly, MOB1A indeed could interact with AMOT, though a little weak, by PUP-IT methods, as seen with the conjugation of AMOT with pupE (See figures below).

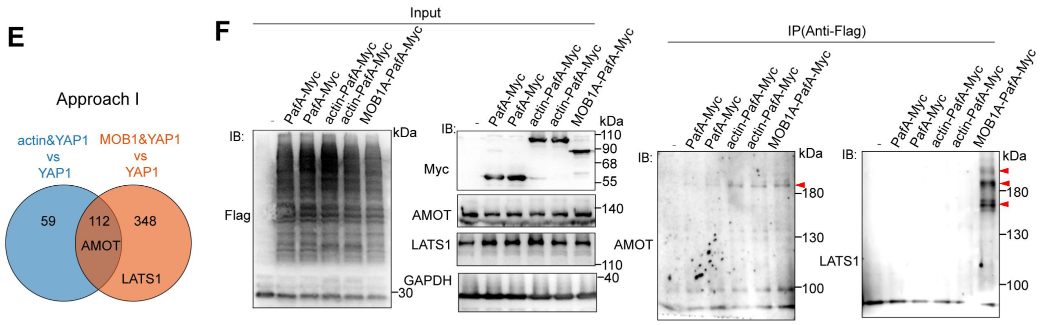

7) For the set of data presented on Fig. 4 I have the same comment as for Fig. 2: I agree that STUPPIT identified α-catenin and LATS1 but the only support for their identification as "intermediary proteins" is the current literature. The data that demonstrate they were conjugated with pup(E) is missing.

We appreciate this key point. We have performed the flag-IP and proved they were conjugated with Flag-TbN-PupE (Fig 3 & S3 Fig).

8) Line 293: what was the rational to select specifically β-catenin and SMAD4?

We selected β-catenin and SMAD4 as prey proteins for STUPPIT, as mentioned in the main manuscript, because they serve as core effector proteins of the Wnt and BMP signaling pathways, respectively. Their pivotal roles in these pathways mean that investigating their potential associations could provide valuable insights into the molecular mechanism underlying the functional counteraction between the Wnt and BMP signaling pathways. This feature makes them ideal prey proteins for validating the ability of STUPPIT to capture novel intermediate interactions between functionally relevant key signaling molecules.

9) Fig. 5C: what are samples 1-6 and 7-12, replicate experiments?

We thank you for your question and apologize for the unclear and incomplete description in the figure legend. Samples 1–6 correspond to 6 replicate experiments from the SMAD4&TbC control group, each conducted in 6 cm dishes. Samples 7–12 correspond to 6 replicate experiments from the SMAD4&β-catenin experimental group, also performed in 6 cm dishes. To ensure sufficient protein enrichment for subsequent analyses, the 6 replicate samples in each group were pooled into 3 biological replicates. This detail has now been added to the relevant figure legend for clarity.

10) Lines 293-304: The performed experiment is not expected to solely identify intermediary proteins of β-catenin and SMAD4 but proteins in close proximity to unknown proteins that previously interacted with SMAD4 and now in complex with β-catenin. Some of them may be intermediary proteins, others not. Therefore it seems at the first glance surprising that 5 out of 5 tested hits are all intermediary proteins. Was this expected, where the hits picked for validation chosen according to special criteria, does it suggest that β-catenin and SMAD4 are in fact part of the same complex?

We thank you for your question. β-catenin and SMAD4 are core proteins of the Wnt and BMP signaling pathways, respectively, and they exhibit functional counteraction in multiple biological contexts—for

example, during the activation of hair follicle stem cells or small intestinal stem cells. While direct interaction between them has not been reported, they may regulate each other's activity indirectly, either through intermediate partners or at the transcriptional level. The candidates RNF20, ERC1, USP7, and TRIM33 were selected to validate their interactions with β-catenin and SMAD4, respectively, based on their appropriate $\log_2$ fold change, p-value, and number of unique peptides—criteria that ensured they were suitable for follow-up testing. We also attempted to validate interactions between FUBP1/ANK3 and β-catenin/SMAD4, but these experiments were unsuccessful. Additionally, we were unable to clone other candidates (e.g., KIF4A and U2SURP) for further testing. Notably, since TRIM33's interaction with these targets has been previously reported, we focused our validation efforts on RNF20, ERC1, and USP7. We used multiple controls (e.g., HA-TbC or GFP-Flag) to further verify their interactions and employed the PUP-IT assay to assess their binding to β-catenin and SMAD4. Our extending experiments showed that ERC1 and USP7 interacted more robustly with β-catenin and SMAD4 (Fig 6), whereas RNF20's interaction with these proteins appeared to be transient (S6 Fig).

Some more general comments:

- *A pulldown with streptavidin is not an immunoprecipation (IP)*

Thank you for pointing out this critical distinction. We have revised the text to use "streptavidin pull-down" instead of "immunoprecipitation (IP)".

- *Doing a t-test analysis based on 3 western blot experiments does not really make statistical sense: just show the individual data points for each replicate + a bar for the mean value (on all figures).*

Thank you for raising this important point regarding the statistical analysis. Given that the experiments were performed in triplicate, we considered that adding individual data points to the bar graphs would provide limited additional information for the reader. Instead, we have now stated in the methods section that all experiments were performed in at least three independent replicates.

- *The description of the MS analysis is not precise enough: MaxQuant yields intensity values, LFQs or iBAQs but does not perform enrichment calculations. Which software was use and with which parameters to perform imputations (if any), calculate fold changes and p-values. The MS data should be deposited in a public repository. These two points are absolutely necessary, especially for a method paper.*

The MS analysis is performed with help of Prof. Xuna Wu in Yunnan University. Protein identification and label-free intensity quantitation (LFQ) were performed by MaxQuant software (version 1.6.17.0). Missing value was imputed by random numbers from a normal distribution applying a downshift of 1.8 times the standard deviation of the global dataset, and a width of 0.3 times the standard deviation. We have supplemented this detail in the Methods section. Additionally, the raw mass spectrometry (MS) data have been deposited in the public repository iProX (project ID: IPX0013559000). Currently, the data deposit is under review by the iProX team and will be made publicly accessible following the publication of this manuscript.

References:

CHO, K. F., BRANON, T. C., RAJEEV, S., SVINKINA, T., UDESHI, N. D., THOUDAM, T., KWAK, C., RHEE, H. W., LEE, I. K., CARR, S. A. & TING, A. Y. 2020. Split-TurboID enables contact-dependent proximity labeling in cells. *Proc Natl Acad Sci U S A,* 117**,** 12143-12154.

MANA-CAPELLI, S. & MCCOLLUM, D. 2018. Angiomotins stimulate LATS kinase autophosphorylation and act as scaffolds that promote Hippo signaling. *J Biol Chem,* 293**,** 18230-18241.
