# Supplementary material for: STUPPIT is a proximity labeling tool for labeling intermediary proteins that bridge two non-interacting proteins
Source: PLoS Biol. 2025 Nov 24;23(11):e3003227. doi: 10.1371/journal.pbio.3003227 (PMC12668627; doi:10.1371/journal.pbio.3003227)
Supplement: Attachment — Submitted filename: Response_to_Reviewers_auresp_3.pdf [file pbio.3003227.s009.pdf]

Response to Reviewers

Reviewer #1: I appreciate that the authors have addressed all my concerns from the previous review. The revisions have substantially improved the clarity and rigor of the work, and I recommend acceptance of this manuscript.

We sincerely appreciate your positive feedback and are delighted to hear that our revisions have successfully addressed all concerns. We are also grateful for your recommendation for acceptance.

Reviewer #2: I would like to congratulate the authors for the efforts they put in the revision of their manuscript which I found significantly improved. I have a few remaining minor redactional comments that I would recommend addressing to be fully supportive for publication.

We thank you for your positive assessment of our revisions and supportive comments regarding publication. We agree that your valuable suggestions have further strengthened the paper, and we have incorporated all of them into the revised manuscript.

Page 14 of the submission file:

"To experimentally verify this in our contex, we further generated two constructs AMOT-Flag-TbN and actin-Flag-TbN. Using these, we assessed the ligase self-labeling and split-TurboID (split at 73/74) activity under two conditions: AMOT-TbN co-expressed with TbC-YAP1, and actin-TbN co-expressed with TbC-YAP1. Consistent with the conclusions from (Cho et al., 2020) in their Fig. 3E, we observed that both the ligase self-biotinylation and TurboID enzymatic activity were far more robust—with significantly stronger signals—in the AMOT-TbN&TbC-YAP1 group than in the actin-TbN&TbC-YAP1 group. This result aligns with the known stronger interaction between AMOT and YAP1 (compared to actin and YAP1), further validating that split-TurboID (73/74) activity directly correlates with the strength of the interaction driving fragment proximity."

Please show and explain the figure corresponding to this experiment (split-TurboID performed on Actin/AMOT vs Actin/YAP1) in the manuscript. The figure can be placed in the supplementary material but it is an important control that provides an important back up to the claim that STUPPIT gives access to information that would not be available with split-turboID.

We appreciate your valuable suggestion. We agree that incorporating this experiment will further strengthen our claim that STUPPIT provides access to information beyond the reach of split-TurboID. Accordingly, we have now included the corresponding data as S1C Fig.

Response to comment #1

The experiment showed on S1B is good but it is not appropriately explained in the main text nor in the legend (one has to do a lot of guesswork to understand the setting). Please explicitly explain in the main text that the experiment shown in S1B corresponds to cells that were co-transfected with plasmids coding for actin-PafA-myc and AMOT-HA.
By contrast, the experiment shown in Fig. 2 corresponds to cells co-transfected with plasmids coding for actin-PafA-myc and HA-TbC-YAP1. Therefore I would also suggest writing in lines 155/159:

As expected, both endogenous (Fig. 2G) and exogenous AMOT (Fig. S1B) were successfully enriched following Flag-IP and exhibited molecular weight laddering detected by anti-AMOT and anti-HA antibody, in contrast to the no-transfection or the PafA-myc transfection control (Fig 2G and S1B Fig).

We thank you for this detailed suggestion. Accordingly, we have now revised the main text to explicitly state the transfection details both in the main manuscript and figure legends for Fig. S1B and Fig. 2.

Response to comment #6
Thank you for performing and showing this control experiment in the revised manuscript. However you do not really explain in the main text why this experiment was performed and I think the general reader will need some help here. Therefore I would recommend adding in lanes 258/260 a few words to explain this control was needed because of the considerable background of split-TurboID (significant assembly of the two Tb fragments even when in the HA-TbC control).

We thank you for this suggestion. To ensure clarity for readers, we have now explicitly explained in the main text that the YAP-TbC control without actin-pafA-myc was essential due to the considerable background signal from the detectable reassembly of the split-TurboID fragments in line 276-279.

In that vein I would also recommend showing (e.g. in the supplementary material) the MS data in AMOT-knockdown cells that you included in your response to comment #1 as this is another proof that AMOT itself served as one of the pupylated proteins that interacted with TbC-YAP1 and thereby reactivated TurboID activity.

We are grateful to the reviewer for this valuable suggestion. In response, we have now included the mass spectrometry data from the AMOT-knockdown experiment as Supplementary Figure 3E-F. Furthermore, the related proteomics dataset has been deposited in the public repository iProX (project ID: IPX0013559000, accession number PXD069286).

Description of the analysis of the MS data:

There are still information missing: which software has been used to calculate enrichments and p-values. What do you define as p-value: for large data sets such as MS data, it is generally accepted that FDR (false discovery rate)-adjusted p-values (aka q-values) should be used. Was this

the case here, if not, why?

We apologize for this oversight in our methodology description and thank you for pointing out this omission. The statistical analysis was performed using Perseus, with enrichment calculated by Fisher's exact test and p-values presented as FDR-adjusted. This information has now been added to the corresponding section of the Methods in line 618-619.

Discussion:

I point two considerations the authors may add in their discussion to help potential users of their new method to optimize their experiments (I leave it to the authors if they want to add these or not):

1) Lines 386/387: one thing that one may consider with the choice of the split-system: both paf and TurboID attach their label (pupE and biotin) to lysine residues. It is therefore possible in certain cases that the first labeling step (pupIT) will saturate all available lysines and make the intermediate protein non-targetable by the second labeling step (biotinylation by TurboID). With this in mind the combination pupIT / split-APEX2 (with APEX2 rather biotinylating on tyrosines) may be worth trying.

2) In the discovery approach as shown on Fig. 5, identification of intermediate proteins is solely based on biotinylation. As discussed by the authors in lines 399/402, biotinylation generated by STUPPIT will label intermediate proteins and prey C but also proximity proteins between the intermediary protein and prey C. It may be worth mentioning that an even more targeted approach would be to first perform a FLAG-IP followed by streptavidin pulldown and MS analysis. This would allow specifically identifying "double labelled" proteins by STUPPIT, which would be even stronger candidates for intermediary proteins

We sincerely thank you for these thoughtful considerations aimed at improving STUPPIT for future users.

1) Regarding the first point, we agree that combining PUP-IT with a tyrosine-targeting system like split-APEX2 is a promising direction to avoid competition for lysine residues. We have incorporated this insightful idea into the Discussion as a potential avenue for future development in line 424-429.

2) We have also considered the sequential purification approach suggested here. While it holds theoretical merit for isolating double-labeled proteins, our practical experience indicates that sequentially combining FLAG-IP and streptavidin pull-downs may not reduce non-specific background, as each step introduces its own cohort of non-specifically bound proteins. This cumulative noise may diminish the signal-to-noise ratio for genuine intermediates. Despite this consideration, we have included a discussion of this approach to provide readers with a comprehensive overview of potential methodological options in line 466-471.
